# Dysregulated CD38 Expression on Peripheral Blood Immune Cell Subsets in SLE

**DOI:** 10.3390/ijms22052424

**Published:** 2021-02-28

**Authors:** Marie Burns, Lennard Ostendorf, Robert Biesen, Andreas Grützkau, Falk Hiepe, Henrik E. Mei, Tobias Alexander

**Affiliations:** 1 Deutsches Rheuma-Forschungszentrum (DRFZ Berlin), a Leibniz Institute, 10117 Berlin, Germany; Marie.Urbicht@drfz.de (M.B.); lennard.ostendorf@charite.de (L.O.); robert.biesen@charite.de (R.B.); Gruetzkau@drfz.de (A.G.); falk.hiepe@charite.de (F.H.); mei@drfz.de (H.E.M.); 2 Department of Rheumatology and Clinical Immunology, Charité–Universitätsmedizin Berlin, Corporate Member of Freie Universität Berlin, Humboldt-Universität zu Berlin and The Berlin Institute of Health (BIH), 10117 Berlin, Germany; 3 Department of Nephrology and Intensive Care Medicine, Charité–Universitätsmedizin Berlin, Corporate Member of Freie Universität Berlin, Humboldt-Universität zu Berlin and The Berlin Institute of Health (BIH), 10117 Berlin, Germany

**Keywords:** CD38, SLE, immune profiling

## Abstract

Given its uniformly high expression on plasma cells, CD38 has been considered as a therapeutic target in patients with systemic lupus erythematosus (SLE). Herein, we investigate the distribution of CD38 expression by peripheral blood leukocyte lineages to evaluate the potential therapeutic effect of CD38-targeting antibodies on these immune cell subsets and to delineate the use of CD38 as a biomarker in SLE. We analyzed the expression of CD38 on peripheral blood leukocyte subsets by flow and mass cytometry in two different cohorts, comprising a total of 56 SLE patients. The CD38 expression levels were subsequently correlated across immune cell lineages and subsets, and with clinical and serologic disease parameters of SLE. Compared to healthy controls (HC), CD38 expression levels in SLE were significantly increased on circulating plasmacytoid dendritic cells, CD14^++^CD16^+^ monocytes, CD56^+^ CD16^dim^ natural killer cells, marginal zone-like IgD^+^CD27^+^ B cells, and on CD4^+^ and CD8^+^ memory T cells. Correlation analyses revealed coordinated CD38 expression between individual innate and memory T cell subsets in SLE but not HC. However, CD38 expression levels were heterogeneous across patients, and no correlation was found between CD38 expression on immune cell subsets and the disease activity index SLEDAI-2K or established serologic and immunological markers of disease activity. In conclusion, we identified widespread changes in CD38 expression on SLE immune cells that highly correlated over different leukocyte subsets within individual patients, but was heterogenous within the population of SLE patients, regardless of disease severity or clinical manifestations. As anti-CD38 treatment is being investigated in SLE, our results may have important implications for the personalized targeting of pathogenic leukocytes by anti-CD38 monoclonal antibodies.

## 1. Introduction

Systemic lupus erythematosus (SLE) is a chronic autoimmune disease characterized by immune responses against nuclear antigens. The immunopathogenesis of the disease is complex and involves genetic, environmental, hormonal, epigenetic, and immunoregulatory factors that may act either sequentially or simultaneously on the immune system [1]. It is assumed that a defect in the clearance of apoptotic cells with accumulation of undigested apoptotic remnants may provoke the first hit in the break of self-tolerance by activating otherwise quiescent autoreactive lymphocytes. The presence of extracellular DNA additionally stimulates plasmacytoid dendritic cells (pDC) via Toll-like receptors, resulting in the production of type I interferons (IFN-I), some of the hallmark cytokines in SLE [1,2,3]. Persistently activated IFN-I signaling pathways, mimicking sustained anti-virus responses, may contribute to SLE pathogenesis by amplifying antigen-specific adaptive autoimmune responses. Upon receiving T cell help, autoreactive B cells are activated, increase their specificity towards nuclear antigens during affinity maturation, and differentiate into class-switched memory B cells and antibody-secreting plasma cells (PC). The newly generated plasma cell precursors detectable in peripheral blood, so-called plasmablasts (PB), migrate to the bone marrow and inflamed organs, where they eventually become long-lived once seeded in dedicated niches and continuously secrete pathogenic autoantibodies for months or even years, thereby contributing to the chronicity of SLE [4]. 

Many of the immune cells that contribute to SLE pathogenesis may express the multifunctional cell-surface protein CD38, either constitutionally or upon stimulation. As an enzyme, CD38 degrades nicotinamide nucleotides like NAD^+^ and both synthesizes and hydrolyzes the second messenger cADPR (cyclic ADP ribose), although the relative contributions of these functions are controversially discussed [5]. While commonly considered an ectoenzyme, CD38 is also detectable within the nucleus and the mitochondrial membrane and as a soluble molecule in the blood [5]. At the same time, CD38 also functions as an activatory receptor on immune cells in the context of cell adhesion, migration, and cytokine secretion [6]. Calcium influx induced by CD38 ligation is also required for dendritic cells and neutrophils to follow chemokine gradients into the lymph node and migrate into inflamed organs [7,8].

Previous studies analyzing the expression of CD38 on leukocytes in SLE indicated increased CD38 expression on different leukocyte subsets compared to healthy controls, such as CD4^+^ and CD8^+^ memory T cells [9,10,11,12] and monocytes [13]. Research in CD38^−/−^ mice found that CD38 deficiency ameliorated the course of pristane-induced lupus [14]. Because CD38 is expressed constitutively at high levels on human antibody-secreting plasmablasts and plasma cells and is inducible on several immune cell subsets upon activation, CD38 has been considered as a potential therapeutic target in autoantibody-driven diseases such as SLE [15], autoimmune encephalitis [16,17], and autoimmune hemolysis [18]. We recently treated two patients with refractory, life-threatening SLE with the anti-CD38 antibody daratumumab, which resulted in substantial clinical and immunological efficacy [11]. Although the CD38-mediated targeting of plasma cells was presumably the most important factor for the observed clinical responses, it remains unclear if and how daratumumab exerts therapeutically relevant effects on other CD38 expressing immune cell subsets. While there are reports of increased CD38 expression in different peripheral immune cell subsets in SLE [9,10,12,13,15], a comprehensive analysis of CD38 expression in the immune cell compartment in SLE is lacking. 

Here, we report the results of a global expression analysis of CD38 on blood leukocytes from two cohorts of SLE patients, using mass and flow cytometry, that confirm and expand previous results, and demonstrate that increased CD38 expression in subsets of innate and adaptive immune cells is a stable and reproducible feature of SLE, largely independent of disease phenotype and severity. 

## 2. Results

### 2.1. Increased CD38 Expression in Major SLE Leukocyte Subsets

**Figure 1:**
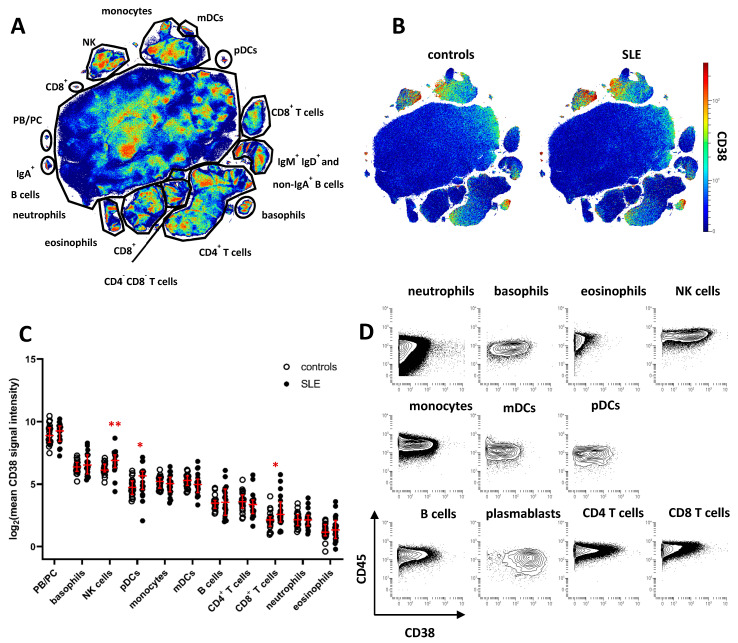
Cell type-specific dysregulation of CD38 expression in patients with SLE. Peripheral blood leukocytes from 20 SLE patients and 20 healthy controls were analyzed by mass cytometry. (**A**) t-SNE map showing all leukocytes analyzed in the study (7.6 × 10^6^ cells). Major cell subsets were annotated. Color indicates cell density. (**B**) CD38 expression across major leukocyte subsets in SLE patients and controls. Concatenated data is shown for both groups. (**C**) Comparison of CD38 expression across major subsets gated in (**A**). Each dot represents the log_2_ of mean CD38 signal intensity (SI) of the indicated subset of one donor. Red lines indicate median values and interquartile range. * indicate significantly different CD38 expression in SLE patients vs. controls revealed by Mann–Whitney testing (* *p* < 0.05; ** *p* < 0.01) (**D**) Contour plot representation of CD38 expression of the indicated leukocyte subsets. Concatenated data of 20 healthy controls are shown.

To characterize CD38 expression across peripheral blood leukocyte subsets and identify potential dysregulation of CD38 in SLE, we analyzed leukocytes isolated from cryopreserved whole blood samples of 20 SLE patients and 20 age- and gender-matched healthy controls (HC). Patient demographics are shown in Table 1. Cell samples were analyzed by mass cytometry, using an antibody panel suitable for analyzing the expression of CD38 in major peripheral blood leukocyte subsets (Supplementary Table S1). Data of CD45^+^ leukocytes were subjected to dimension reduction by opt-SNE [19], and major cell subsets were annotated according to the expression of lineage-defining cell-surface markers on the resulting t-SNE map (Figure 1A and Supplementary Figure S1). CD38 expression was projected onto the t-SNE maps of concatenated data of SLE or HC samples (Figure 1B). Highest CD38 expression was found on PB/PC (mean signal intensity HC, 572; SLE, 586), followed by NK cells (HC, 79; SLE, 132) and basophils (HC, 89; SLE, 119), monocytes (HC, 38; SLE, 35), and plasmacytoid and myeloid dendritic cells (pDC and mDC) (HC, 30; SLE, 52, and HC 40; SLE 38, respectively). On average levels, CD38 was absent from or weakly expressed by most T and B cells, eosinophils, and neutrophils. Based on the mean CD38 expression of each subset, we found significantly increased CD38 expression in SLE samples on NK cells, pDC, and CD8^+^ T cells (1.7-, 1.7-, and 2.5-fold increase, respectively, Figure 1C). Since subset-specific CD38 expression varied among both SLE patients and HC, we determined the coefficient of variation (CV) for CD38 expression levels in the leukocyte subsets identified in Figure 1A. Highest CV differences between SLE and HC were identified for CD8^+^ T cells (mean CV, HC, 61%; SLE, 131%), B cells (HC, 44%; SLE, 95%), eosinophils (HC, 41%; SLE 84%), CD4^+^ T cells (HC, 60%; SLE 94%), and mDC (HC, 35%; SLE, 68%). In addition, we found evidence of varying CD38 expression on the single-cell level in almost all leukocytes subsets (Figure 1B,D), prompting us to analyze the variability of CD38 expression in greater detail.

### 2.2. CD38 Expression by NK Cells and Myeloid Immune Cells Is Associated with an Activated Phenotype

Based on our initial finding of increased expression of CD38 in SLE NK cells, but heterogeneous detection of CD38 on NK cells at the single-cell level, we analyzed whether NK cell CD38 expression levels were associated with other phenotypical features of NK cells. Among NK cells, high expression of CD38 was associated with increased expression of CD11c, and CD38^++^ NK cells comprised most HLA-DR-expressing NK cells in HC and SLE patients (Supplementary Figure S2). Together, these data suggest that high CD38 expression could be a feature of activated NK cells, and the increased CD38 expression by NK cells may indicate enhanced activation of circulating NK cells in SLE. Similar observations were made for basophils, mDC, pDC, and monocytes. For example, CD38^+^ mDC co-expressed Syk, HLA-DR, and CD11c at higher levels, and CD38^+^ pDC co-expressed increased levels of Syk and HLA-DR compared to their CD38^−/low^ counterparts (Supplementary Figure S2). In monocytes, CD38 expression was significantly associated with expression of SIGLEC-1 (*p* <0.0001), a marker associated with IFN-I activity [3,20]. 

### 2.3. Increased Expression of CD38 on Distinct Subsets of Peripheral Blood B Cells in SLE

**Figure 2:**
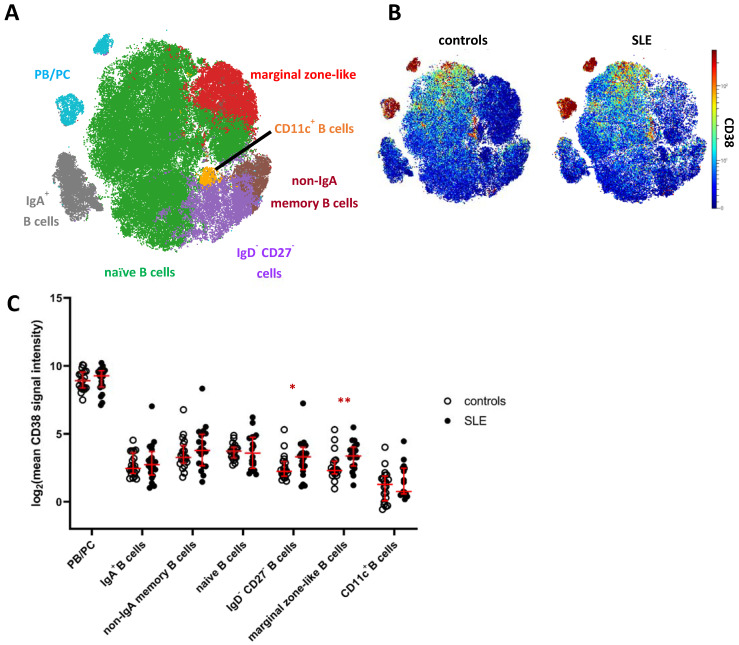
CD38 expression across B cell subsets in patients with SLE. (**A**) t-SNE map showing clusters of CD19^+^ B cells generated by FlowSOM from the mass cytometry dataset. Clusters comprising naive B cells (green) and PB/PC (blue) were merged for further analyses (Supplementary Figure S3). (**B**) CD38 expression across B cells in SLE patients and controls. Both plots show concatenated data of the respective group. (**C**) Comparison of CD38 expression across major clusters as depicted in (**A**). Each dot represents the log_2_ of mean CD38 signal intensity (SI) of the indicated subset of one donor. Red lines indicate medians and interquartile ranges. * indicate significantly different CD38 expression in SLE patients vs. controls revealed by Mann–Whitney testing (* *p* < 0.05; ** *p* < 0.01).

Next, we analyzed the mass cytometry data of CD19^+^ B cells, including HLA-DR^high^ plasmablasts and HLA-DR^low^ plasma cells [21], by FlowSOM clustering and subsequent hierarchical metaclustering, based on markers expressed by B cells and omitting CD38 (Supplementary Table S1). We obtained ten individual B cell clusters, including two IgD^+^IgM^+^ naive B cell clusters, IgA^+^ and IgA^−^ memory B cells, CD11c^+^ B cells, CD27^+^IgD^+^IgM^+^CD1c^+^ marginal zone-like B cells, CD27^−^IgD^−^ B cells, and three clusters of PB/PC distinguished by differential expression of IgA and HLA-DR (Supplementary Figure S3A,B). Naive B cell clusters (c1, c3) and clusters comprising PB/PC (c8, c9, c10) were merged for downstream analyses (Figure 2A, Supplementary Figure S3A). Confirming our results from the global analysis, PB/PC showed the highest expression of CD38 among B cells, followed by naive and memory B cell clusters showing overall lower average expression of CD38 (Figure 2A,B). The lowest mean CD38 expression in the B cell lineage was detected on CD11c^+^ B cells, which are linked to chronic inflammation [22,23]. We again tested for differences in the expression of CD38 in SLE vs. HC and detected an increased mean CD38 expression on CD27^−^/IgD^−^ B cells (2.2-fold increase in SLE) and marginal zone-like B cells (1.6-fold increase), the latter showing the *p* value between patients and controls. Consistently, we detected significantly increased frequencies of CD38^hi^ and CD38^int^ B cells among marginal zone-like B cells (3.3-fold, *p* = 0.01 and 2.0-fold, *p* = 0.003) and of CD38^hi^ cells among CD27^−^IgD^−^ B cells (2.6-fold, *p* = 0.05) in SLE patients, but not among other B cell clusters (Supplementary Figure S3D,E). Since targeting of PB/PC is one major rationale for CD38-directed treatment in SLE, we analyzed whether subsets of PB/PC expressed similar levels of CD38, and hence stratified IgA^+^ and IgA^−^ PB/PC, and HLA-DR^high^ PB vs. HLA-DR^low^ PC. In all four subsets, we observed the same trend of increased CD38 expression in SLE patients vs. HC detectable in total PB/PC (Figure 1C), yet not associated with statistical significance (Supplementary Figure S3E). When SLE and HC samples were combined, we did, however, find that IgA^−^ PB/PC (that is, IgG^+^ and IgM^+^ PB/PC) expressed higher levels of CD38 compared to their IgA^+^ counterparts (1.2-fold, *p* = 0.07) and that mean CD38 expression levels were higher on HLA-DR^high^ PB compared to HLA-DR^low^ PC (1.4-fold increase, *p* = 0.01).

### 2.4. Circulating CD4^+^ and CD8^+^ Memory T Cell Subsets Express Increased Levels of CD38 in SLE

**Figure 3:**
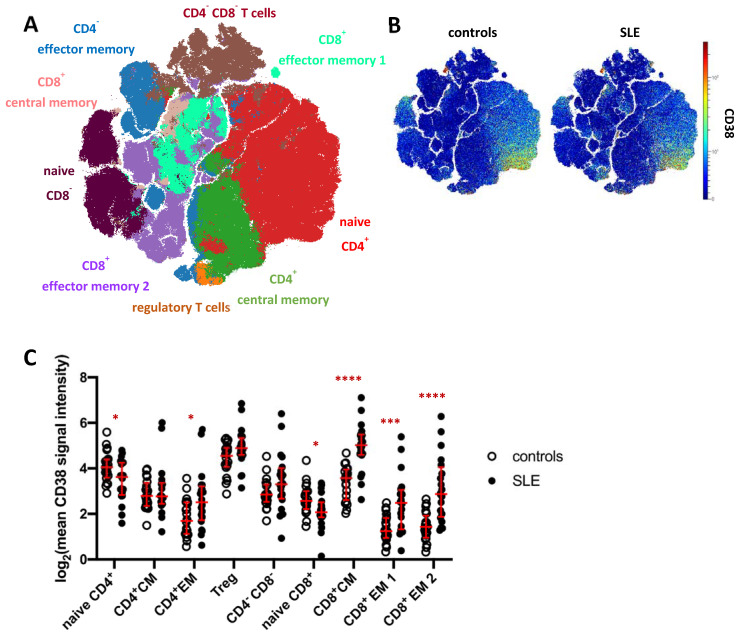
CD38 expression across T cell subsets in patients with SLE. (**A**) t-SNE map showing T cell clusters obtained by FlowSOM clustering from the mass cytometry dataset. Major T cell subsets are annotated. Clusters comprising CD4^−^CD8^−^T cells (brown), CD4^+^ effector memory T cells (blue), and CD8^+^ effector memory subsets (turquoise) were merged for downstream analyses (Supplementary Figure S4A). (**B**) CD38 expression across T cells in SLE patients and controls. (**C**) T cell clusters depicted in (**A**) were analyzed for their CD38 expression. Each dot represents the log_2_ of mean CD38 signal intensity (SI) of the indicated subset of one donor. Red lines indicate medians and interquartile ranges. * indicates significantly different CD38 expression in SLE patients vs. controls revealed by Mann–Whitney testing (* *p* < 0.05; *** *p* < 0.001; **** *p* < 0.0001).

To analyze the CD38 expression pattern and to address potential changes in SLE, we subjected total CD3^+^ T cells (merged from all T cell subsets in Figure 1A) to FlowSOM clustering and subsequent hierarchical metaclustering to obtain fifteen T cell clusters, comprising five CD4^−^ CD8^−^ T cell clusters, three CD8^+^ effector memory (EM) subsets, two CD4^+^ effector memory clusters and clusters representing naïve CD4^+^ and CD8^+^ T cells, regulatory T cells (Treg), as well as CD4^+^ and CD8^+^ central memory (CM) T cells (Figure S4). CD4^−^ CD8^−^ T cell clusters (c6, c8, c9, c10, c11), CD4^+^ effector memory clusters (c4, c5), and CD8^+^ effector memory subsets (c7, c14) were merged for further analysis (Figure 3A). Global CD38 expression for concatenated SLE patient and HC data was projected onto a t-SNE dimension reduction plot (Figure 3B), confirming that CD38 was variably expressed across individual T cells and T cell clusters. In HC, naïve CD4^+^ T cells showed higher average expression of CD38 compared to naïve CD8^+^ T cells (*p* < 0.0001, 2.7-fold) and to CD4^+^ central and effector memory T cell clusters, respectively (*p* < 0.001). The same trend was observed for CD8^+^ effector memory vs. naïve T cells. Of all T cell subsets analyzed in this study, Tregs showed the highest average CD38 expression. 

CD38 expression by T cell clusters was found to be strongly dysregulated in SLE (Figure 3C). In line with increased CD38 expression in B cell and innate immune cell subsets, significantly increased CD38 expression was evident in CD4^+^ and CD8^+^ memory T cells. The largest differences were found in CD8^+^ central memory and effector memory subsets (3.4-fold, 3.1-fold, and 4.9-fold SI, respectively). CD4^+^ effector memory T cells also showed increased CD38 expression in SLE patients compared to controls (2.4-fold SI). Regulatory T cells showed a tendency towards higher CD38 levels in SLE, which did, however, not yet reach statistical significance. In contrast, naïve CD4^+^ and CD8^+^ T cells showed reduced CD38 expression in SLE compared to HC (both 1.4-fold, *p* = 0.04 and *p* = 0.02, respectively). 

In accordance with previous works, we observed that CD38 expression was inhomogeneous in clusters of naive and memory T cells (Supplementary Figure S4C), and we determined frequencies of CD38^−/low^, CD38^int^, and CD38^hi^ cells among the different clusters in SLE patients and HC to account for this heterogeneity. Except for naïve CD4^+^ T cells and Tregs comprising comparably high average frequencies of CD38^int^ and CD38^hi^ cells of 30% and 13%, and 13% and 16%, respectively, all T cell clusters comprised an average of 80% or more CD38^−/low^ cells, indicating that CD38 is selectively expressed by relatively few T cells in peripheral blood. In SLE, we detected significantly increased frequencies of CD38^int^ and CD38^hi^ cells subsets in CD4^+^ and CD8^+^ effector memory subsets, CD8^+^ central memory, Tregs, and CD4^−^CD8^−^ T cells (Supplementary Figure S4). Thus, CD38 was variably expressed by T cells in the peripheral blood, and CD38 expression allowed for the separation of CD38^int^ and CD38^hi^ expressing T cell subsets. Except for naïve CD4^+^ T cells, CD38 expression marked minor fractions of the different T cell subsets. CD38 expression, the presence of CD38-expressing T cells in the blood, or both, were dysregulated in SLE in naive and memory T helper and cytotoxic T cells, with most striking differences in CD8^+^ memory T cells. 

We noted that some SLE patients, but not HC, showed distinctly high expression of CD38 in memory T cell subsets, indicating that beyond the detection of significantly increased CD38 expression in various memory T cell and other subsets in the entire SLE group, CD38 expression by T cells may strongly vary from patient to patient.

In summary, mass cytometric profiling indicated increased CD38 expression levels in almost all major peripheral blood immune cell lineages in SLE on a global level, which were attributable to increased expression levels on individual, mostly activated immune cell subtypes when analyzed on the single-cell level.

### 2.5. CD38 Expression Is Increased on Monocyte, NK Cell, and B Cell Subsets in an Independent SLE Cohort Flow Cytometry Cohort

**Figure 4:**
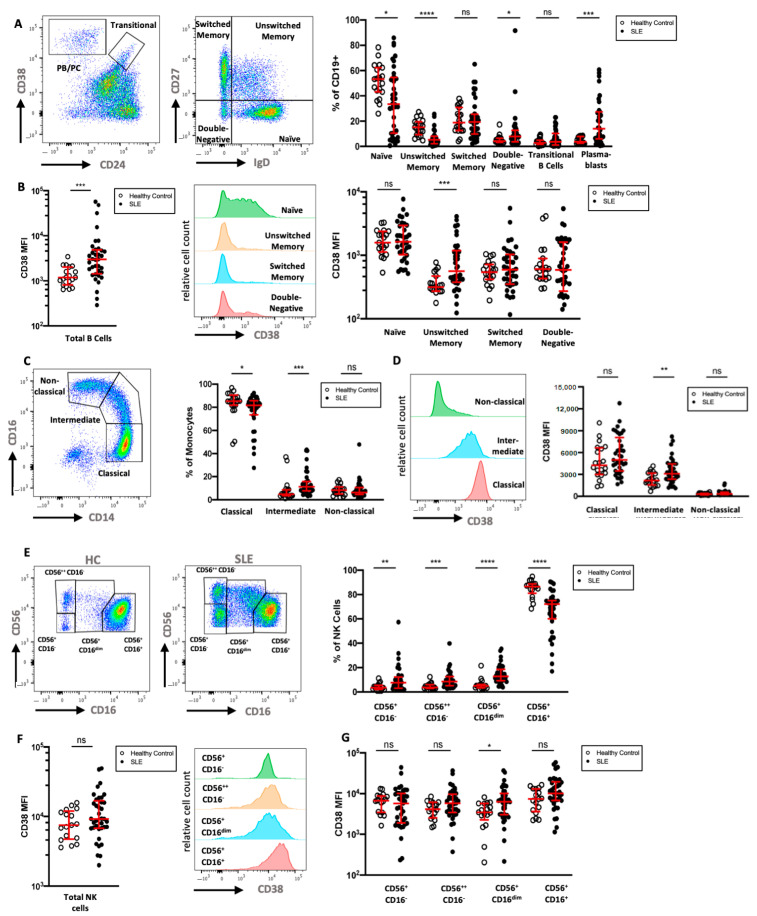
Flow cytometric analysis of CD38 expression in B cells, monocytes, and NK cells (**A**) Gating of CD19^+^ B cells in a HC, with separation of plasmablasts (CD24^−^CD38^++^) and transitional B cells (CD24^++^CD38^++^). B cells that were neither plasmablasts nor transitional B cells were divided into naive (IgD^+^CD27^−^), unswitched memory (IgD^+^CD27^+^), switched memory (IgD^−^CD27^+^), and double-negative B cells (IgD^−^CD27^−^). Relative frequencies of B cell subsets in 36 SLE patients and 19 HC are shown. (**B**) Comparison of CD38 expression on all B cells and on the previously defined B cell subsets in SLE and HC. (**C**) Gating and relative frequencies of classical (CD14^++^CD16^−^), intermediate (CD14^++^CD16^+^), and non-classical (CD14^+^CD16^++^) monocytes. (**D**) CD38 expression on monocyte subsets in a representative HC and comparison of CD38 expression on monocyte subsets between HC and SLE patients (**E**). Representative gating of CD56^+^CD3^−^ NK cell subsets into four subsets in one HC and one SLE patient. (**F**,**G**) Comparison of CD38 expression on all NK cells and on the previously defined NK cell subsets. Mann–Whitney test of 19 HCs and 36 SLE patients. ns, not significant, *p* >= 0.05; * *p* < 0.05; ** *p* < 0.01; *** *p* < 0.001; **** *p* < 0.0001; MFI: median fluorescence intensity.

To extend the mass cytometry results, we analyzed the CD38 expression of key leukocyte subsets in a second cohort of 36 SLE patients by multicolor flow cytometry and compared the data to that of 19 HC (Table 1). We analyzed six subsets of CD19^+^ B cells, in particular CD24^−^CD38^++^ PB/PC, CD24^++^CD38^++^ transitional B cells, and B cells that were neither PB/PC nor transitional B cells, which were further divided according to their expression of IgD and CD27 (Figure 4A). As previously described [24,25,26], SLE patients showed elevated frequencies of IgD^−^CD27^−^double-negative B cells, transitional B cells, and PB/PC, but decreased frequencies of IgD^+^CD27^−^ naive and IgD^+^CD27^+^ unswitched memory B cells. Naive B cells in HC showed intermediate expression of CD38, while most memory and IgD^−^CD27^−^ B cells displayed low if any expression of CD38. Similar to the cohort analyzed by mass cytometry, marginal zone-like IgD^+^CD27^+^ B cells had significantly higher expression levels of CD38 compared to HC (2.8-fold increase), and CD38 expression on IgD^−^CD27^+^ class-switched memory B cells was increased in SLE (1.5-fold increase, Figure 4B). While not reaching statistical significance, the relative difference in mean expression of CD38 was comparable and confirmatory to the mass cytometry results.

Next, we analyzed monocyte subsets in more detail. In particular, classical (CD14^+^CD16^−^), intermediate (CD14^+^CD16^dim^), and non-classical (CD14^dim^CD16^+^) monocytes were investigated (Figure 4C). SLE patients showed higher relative frequencies of intermediate monocytes and lower frequencies of classical monocytes compared to HC. We found that monocytes express intermediate levels of CD38, and that the expression of CD38 was highest on classical monocytes and lower on intermediate and non-classical monocytes (Figure 4D). There was no significant difference in the CD38 expression of total monocytes when comparing SLE patients and HC; however, significantly increased CD38 expression was evident on intermediate monocytes in SLE (1.5-fold increase). 

While most circulating NK cells expressed a CD56^+^CD16^high^ phenotype in SLE patients, we found increases in the fractions of CD56^low^CD16^−^, CD56^high^CD16^−^, and especially of the CD56^+^CD16^dim^ NK cells, a phenotype that has been observed in NK cells after engagement with target cells [27] (Figure 4E). CD38 expression was uniformly high on NK cells of both patients and HC; however, SLE patients had a larger variation in median CD38 expression (Figure 4F). While three out of four NK cell subsets showed a trend towards higher CD38 expression in SLE patients, only the CD56^+^CD16^dim^ subset had significantly increased CD38 levels (2.2-fold increase, Figure 4G). 

Given the significant difference in age and sex distribution between HC and SLE patients in these cohorts (Table 1), we analyzed the correlation between age and the CD38 expression levels on immune cell subsets, in which we observed significant differences in CD38 expression. No significant correlation (Spearman correlation, *p* > 0.05, |r| < 0.4) was found in this limited sample size, neither for HC nor for SLE patients. Similarly, CD38 expression levels did not significantly differ between male and female donors. In summary, these results expand the mass cytometry results with consistent increases in CD38 expression on subsets of B cells and NK cells in an independent cohort of SLE patients.

### 2.6. CD38 Expression Correlates between Individual Immune Cell Subsets

**Figure 5:**
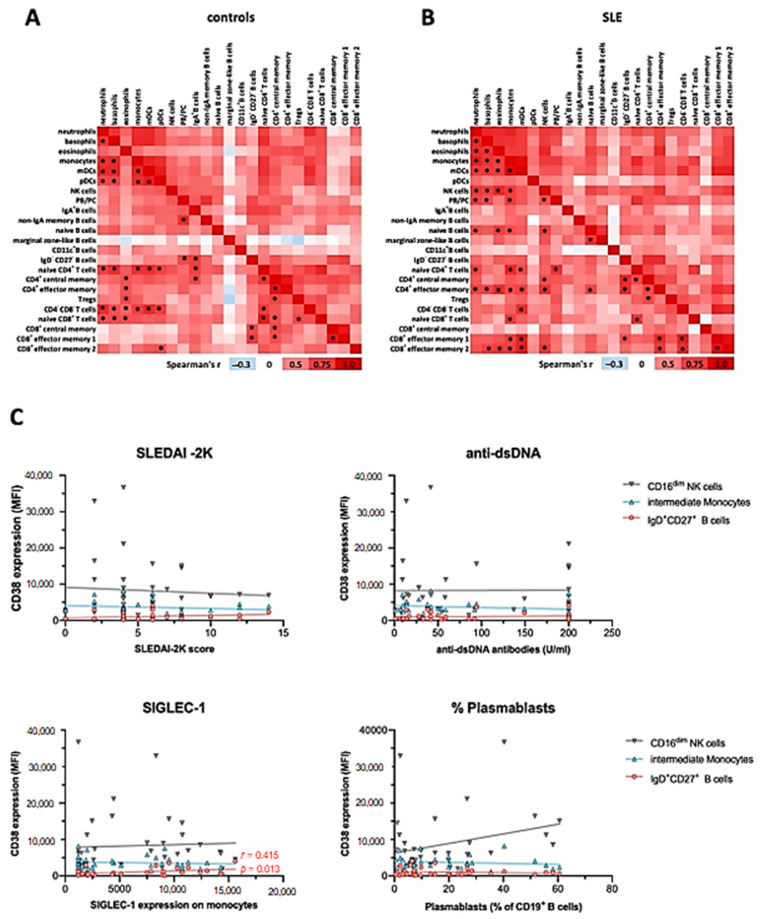
Correlation of CD38 expression levels across major immune cell subsets and with clinical activity. Mean CD38 SI values of immune cell subsets identified in Figure 1, Figure 2 and Figure 3 were analyzed using Spearman correlation. Red color indicates positive correlation (*r* > 0), white indicates no correlation (*r* = 0), and blue color indicates negative correlation (*r* < 0). * indicates significant correlations with a *p* value of < 0.001. (**A**) Data of 20 healthy controls. (**B**) Data of 20 SLE patients. (**C**) Spearman correlation of the disease activity score SLEDAI-2K, the immunological disease activity markers dsDNA antibody levels, SIGLEC-1 expression on monocytes, and the percentage of plasmablasts among CD19^+^ B cells with the CD38 expression on CD16^low^ NK cells, CD14^++^CD16^+^ monocytes, and IgD^+^CD27^+^ B cells, based on the flow cytometric analysis of 36 SLE patients.

To assess the potential correlation of CD38 expression in different immune cell subsets from the mass cytometry dataset, we performed Spearman correlation of mean signal intensity (SI) values across the cell types and subsets identified, separately for controls and SLE patients (Figure 5). We found that CD38 expression levels were either positively or not correlated with each other across the different immune cell types. Inverse correlations were rarely observed and did not reach statistical significance. In HC, we observed 45 positive correlations with *p* < 0.001 (Figure 5A), while we found 56 in SLE (Figure 5B).

In controls, CD38 expression levels on myeloid lineages, except for eosinophils, were widely correlated (Figure 5A). Furthermore, CD38 expression levels on naïve CD4^+^ and CD8^+^ T cells, as well as CD4^−^CD8^−^ DN T cells, correlated with those on innate immune cells in many instances, especially with subsets that robustly express CD38, that is, basophils, monocytes, and pDC. Additionally, we observed correlations between CD38 levels of plasmablasts and other B cell subsets, as well as CD4^+^ and some CD8^+^ T cell subsets. On the background of overall positively correlated CD38 expression, marginal zone-like B cells were a notable exception, inasmuch as r values were consistently smaller compared to the overall r values observed in this analysis. In SLE patients, we observed a profound reconfiguration in the correlation landscape of CD38 expression (Figure 5B). Different from controls, CD38 expression by pDC did not significantly correlate with CD38 levels expressed by other immune cell subsets, while eosinophil CD38 expression was significantly correlated with CD38 levels of other myeloid cell lineages. Furthermore, NK cell CD38 levels showed multiple positive correlations with all innate cell subsets except DC and with memory T cell subsets. PB/PC CD38 levels were associated with those of neutrophils, basophils, monocytes, and naïve CD4^+^ T cells, again, only in patients with SLE.

Taken together, this analysis suggests that CD38 expression by peripheral blood immune cells is remarkably coordinated, especially across innate cell types and T cells, but also with notable examples spanning different hematopoietic lineages. SLE patients exhibit a reconfigured correlation landscape of CD38 expression, suggesting that CD38 expression, or the abundance of CD38^+^ cells in different lineages is at least in part regulated by the same or interrelated factors.

Finally, to assess whether SLE patients and HC could be distinguished by their CD38 expression profile, we performed dimension reduction using multidimensional scaling (mds) based on mean CD38 expression of all 40 donors analyzed by mass cytometry across all immune cell types and subsets (Supplementary Figure S5B). SLE patients and controls were indeed distinguishable by their immune cell CD38 expression profiles, with SLE patients and HC occupying mostly distinct areas of the mds plot. SLE patients showed a more diverse pattern of CD38 expression levels than controls, with some patients grouping closer to HC, while others were clearly separated. Notably, SLE outliers (exceeding the value of 5 in mds_1) are the same that showed extraordinarily high CD38 expression in memory T cell subsets, indicating that T cell CD38 expression is a major determinant of patient heterogeneity related to CD38 expression. 

In summary, intraindividual correlation of CD38 expression levels by different leukocyte subsets was common in HC, with clusters of the statistically robust correlations between subsets of shared lineages. While there was still a high degree of correlation between the CD38 expression in different immune cell subsets in SLE, some correlations were selectively found in SLE patients, such as among T cell subsets, while on some cell types, such as pDCs, CD38 expression was less often significantly correlated with other subsets.

### 2.7. CD38 Expression Levels on Immune Cell Subsets Does Not Correlate with Severity or Clinical Manifestations of SLE

Consistently, and although CD38 has been described as an activation marker on immune cells [28], the increased levels of CD38 on the different subsets in SLE did not correlate with clinical activity as measured by the Systemic Lupus Erythematosus Disease Activity Index 2000 (SLEDAI-2K) (Figure 5C). Likewise, we did not observe correlations of CD38 expression patterns with serum levels of anti-double-stranded DNA (dsDNA) antibodies or complement consumption. In addition, we did not find significant correlations of CD38 expression levels with the presence of specific organ manifestations such as nephritis, mucocutaneous, musculoskeletal symptoms, or use of immunosuppressive treatments. Given the potential role of IFN-I in upregulating CD38 [29], we further analyzed the expression of monocytic CD169 (SIGLEC-1), an established surrogate marker for IFN-I activity [20], in the context of the CD38 expression profiles. While SIGLEC-1 expression in monocytes from SLE patients was significantly increased in both SLE cohorts, and SIGLEC-1^+^ monocytes robustly expressed CD38 (Supplementary Figure S2B), there was no correlation with average CD38 expression on total monocytes or intermediate monocytes across the patients, indicating that a stimulus independent from IFN-I may have induced the increased CD38 expression in SLE intermediate monocytes. However, the CD38 expression on marginal zone-like B cells mildly correlated with SIGLEC-1 expression on monocytes (*r* = 0.415, *p* = 0.013). Taken together, although the CD38 expression levels in SLE were increased on different cell types, CD38 expression did not correlate with clinical severity, serologic activity, or individual disease manifestations.

## 3. Discussion

Motivated by the potential therapeutic use of CD38-targeting antibodies for the treatment of SLE, this study dissected the expression profiles of CD38 in peripheral blood immune cells and described dysregulated CD38 expression in SLE patients compared to healthy controls. In accordance with previous reports [9,10,12], we found that CD38 was variably expressed on all immune cell types, with cell-type specific expression levels peaking in PB/PC. CD38 showed inhomogeneous expression profiles in innate and even more so in adaptive immune cell subsets, ranging from lack of expression to very high levels. This heterogeneity was detectable at levels of low- and high-resolution analyses of different cells types, indicating that the capacity to express CD38 is not restricted to certain immune cell lineages. Initial studies already indicated increased CD38 expression levels on peripheral blood leukocyte cells isolated from SLE patients [9,10,11,12,15]. Our data confirm and extend these findings. In particular, we found CD38 expression levels in SLE significantly increased on circulating plasmacytoid dendritic cells, CD14^++^CD16^+^ intermediate monocytes, CD56^+^ CD16^dim^ natural killer (NK) cells, marginal zone-like IgD^+^CD27^+^ B cells, and subsets of central and effector memory CD4^+^ and especially CD8^+^ T cells. However, although the CD38 expression on different immune cell subsets showed high intraindividual correlations and leukocyte CD38 expression allowed to segregate most SLE patients from HC, we did not identify significant correlations between CD38 expression levels and disease severity or clinical manifestations, suggesting that increased CD38 expression by immune cells is a static feature of SLE, mechanistically or timely unrelated to acute inflammation and clinical flares. However, CD38 expression profiles, as generated in the present study, integrate potentially superimposed regulation of CD38 expression by individual cells and the emergence and homeostasis of cell subsets expressing different levels of CD38 in the blood. Both potential SLE- or inflammation-related induction of CD38 expression [13,15] and overabundance of constitutively CD38-expressing cells such as PB/PC, being established biomarkers for disease activity in SLE [24], have been described in active SLE and cannot be reliably distinguished from each other. Irrespective of what may cause dysregulation of CD38 in immune cells in SLE, the magnitude of CD38 expression across the different cell types may not be a suitable biomarker candidate of the SLE disease activity and phenotype. Instead, the ability to identify patients with increased CD38 expression or abundance of CD38-expressing cells might be relevant in the context of emerging CD38-directed treatments with approved or preclinical candidate compounds, including CD38 CAR T cells, daratumumab, isatuximab, GBR 1342, TAK169, and TAK079. In that regard, our previous report of two SLE patients who underwent anti-CD38 targeted treatment with the monoclonal antibody daratumumab provided the first insight into consequences of CD38-targeting antibodies on the immune system outside malignant conditions [11]. We observed overall stable counts of the major blood leukocyte subsets, except for NK cells and pDC, both robustly expressing CD38, which transiently decreased in circulation after anti-CD38 treatment. However, more intricate effects of daratumumab treatment may occur, such as interference with B cell and T cell maturation in the bone marrow and thymus, where CD38 is expressed in pro-B cells, pre-B cells, and transitional B cells, as well as CD4^+^CD8^+^ double positive thymocytes [30,31,32,33] in addition to memory plasma cells. In peripheral blood, high expression of CD38 has been suggested as a marker of immunosuppressive, so-called regulatory B cells [34], while pro-inflammatory GM-CSF-producing B cells express low levels of CD38 [35]. CD38 on T cells has been reported to be expressed both on activated, proinflammatory T cells [36] as well as on regulatory T cells [37].

The increased expression of CD38 on SLE marginal zone-like B cells and also CD27^+^IgD^−^ switched memory B cells was previously unrecognized. This may result from expansions of CD20-expressing plasmablast precursors that already express high levels of CD38 [38]. In fact, a small population of human blood CD20^+^CD27^+^CD43^+^IgD^+/−^ B cells, which includes such PB precursors as well as a unique population exercising function akin to murine B1 cells [38,39], was found to be increased in SLE [40], and may contribute to the increase of median CD38 expression in CD27^+^ B cells in SLE.

Taken together, our data indicate CD38-targeting treatments are expected to have a wide cellular range of action beyond the targeting of PB/PC, preferentially acting on cells expressing high to very high levels of CD38, and exerting immunomodulation by distinct mechanisms, i.e., by depletion of certain cell types, and non-depleting binding to cell-surface CD38 on others. Vice versa, effects of anti-CD38 monoclonal antibodies on CD38^−/low^ cells cannot be excluded, since they might be secondary to the modulation or depletion of primary target cells. Overall, the effects of CD38-targeted therapies on the immune system will require further investigations, as the effect on the equilibrium of pro- and anti-inflammatory cells is not obvious. Future studies also need to incorporate the distribution of CD38 on immune cells in inflamed tissues, such as skin and kidney in SLE patients.

The individual cell subsets with increased CD38 expression deserve follow up studies to fully explore the expression profile of CD38 across cell subsets, activation, and differentiation states. In particular, it needs to be investigated whether the group of SLE patients characterized by high CD38 immune cell levels (i) maintain this phenotype over the course of disease or (ii) have a different prognosis or long-term outcome compared to those without dysregulated CD38 expression, and (iii) whether this is associated with a certain genetic background. The mechanism of action of CD38-directed therapies on the different cell types beyond depletion requires exploration, including the modulation of its enzymatic activity. As CD38 is a proposed treatment target for SLE, identification of patients with profound increases in CD38 expression pre-treatment could serve as a predictor for treatment responsiveness and thus advance personalized treatments in SLE. 

It would be interesting to determine the factors controlling the differential CD38 expression on immune cells and the subset-specific increase of CD38 expression in SLE. Previous reports indicated that a large range of activatory stimuli may modulate the expression of CD38 on different immune cell subsets. Particularly, type I interferons, hallmark cytokines of SLE pathogenesis, were previously shown to induce CD38 [29]. Other known inducers of CD38 expression include interferon gamma [41], tumor necrosis factor (TNF) [42], and LPS [13]. Nevertheless, we found that CD38 expression on different CD38 subsets only poorly correlated with levels of the established interferon surrogate parameter SIGLEC-1 [3], indicating that additional stimuli likely modulate the expression of CD38 in SLE. On pDC, CD38 is inducible by TLR agonists, such as influenza virus, and, when treated with anti-CD38 in vitro, the capacity of pDC to produce TNFa and IFNa is largely abrogated [43]. Other cells, such as antibody-secreting cells (plasmablasts and plasma cells), constitutively express high levels of CD38. The fact that CD38 levels on separate immune cell subsets showed moderate correlations in general but multiple subgroups of high intraindividual correlation, indicates that a combination of different stimuli as well as differential receptor expression may be responsible for the up-regulation of CD38 on certain cell types. 

A potential caveat for the interpretation of CD38 expression levels obtained from cytometric assays is the reported presence of anti-CD38 autoantibodies in SLE patients that could potentially downregulate CD38 or inhibit the binding of detection antibodies. Interestingly, a previous study found that endogenous anti-CD38 antibodies negatively correlated with disease activity, which could indicate that endogenous anti-CD38 activity confers a protective effect in SLE [10]. Previous research on the role of NK cells in the pathogenesis of SLE focused on the relative hyporeactivity and impaired cytotoxicity in NK cells [44] and reported the relative increase in CD56^bright^ NK cells [45], which we reproduced in this report. Additionally, we report an increase in the frequency of CD16^dim^ NK cells in SLE, a phenotype that has been linked to a post-activation state of NK cell [27]. The potential role of these cells in the SLE pathogenesis, as well as their value for diagnosis and prognosis, are important aspects for future studies.

In conclusion, we identified a widespread dysregulation of CD38 expression in SLE that was found over a variety of leukocyte subsets in the peripheral blood. CD38 expression highly correlated over different leukocyte subsets within individual patients, but was heterogenous within the population of SLE patients. Future studies will be needed to identify the mechanisms that influence CD38 expression in SLE as well as the pathogenic role of cells with increased CD38 expression, especially in light of anti-CD38 monoclonal antibodies being an emerging plasma cell-targeting therapy in SLE.

## 4. Materials and Methods

### 4.1. Patient and Control Blood Samples

Initially, we recruited 20 SLE patients from the Charité–Universitätsmedizin Berlin, Department of Rheumatology and Clinical Immunology. These patients were analyzed using mass cytometry, compared to 20 age- and gender-matched healthy controls. In an additional cohort, 36 SLE patients and 19 healthy controls were included for the flow cytometric analysis. A subset of 10 patients was included in both cohorts. Patient characteristics are summarized in Table 1. SLE patients were diagnosed according to the 2019 EULAR/ACR classification criteria for SLE [46]. Clinical manifestations, the SLEDAI-2K [47], and immunosuppressive medication were recorded by the treating physician. Established markers of immunological activity such as complement factor levels, autoantibody titers, and SIGLEC-1 expression on monocytes [20] were routinely analyzed by the local laboratory (Labor Berlin).

### 4.2. Cryopreservation of Whole Blood Samples

Heparinized whole blood samples were cryopreserved using Proteomic Stabilizer (Smart Tube Inc., San Carlos, CA, USA) as indicated by the manufacturer within 30 min after phlebotomy and stored at −80 °C. On the day of processing, samples were thawed in a stirred water bath at 10 °C and incubated with 5 mL of thaw-lyse buffer (Smart Tube Inc.) for 10 min at room temperature in 15 mL centrifuge tubes. Cells were centrifuged at 700× *g* for 5 min and the erythrocyte lysis step was repeated with 25 mL of thaw-lyse buffer for 5 min at room temperature in 50 mL centrifuge tubes. Peripheral blood leukocytes were washed twice with cell staining medium (1 × PBS made from 10 × PBS ((Rockland Immunochemicals, Gilbertsville, PA, USA) using MilliQ water, supplemented with 0.5% (*w/v*) BSA (PANBiotech, Aidenbach, Germany) and 0.02% sodium azide (Sigma-Aldrich, St. Louis, MO, USA)). Cell counts were determined volumetrically using a MACSQuant Analyzer 10 (Miltenyi Biotec, Bergisch-Gladbach, Germany). Prior to further processing in 5 mL polystyrene tubes (Corning, Corning, NY, USA), cell counts were adjusted to 1.6 × 10^6^ cells per sample.

### 4.3. Mass Cytometry

Samples were barcoded for 20 min using a five-choose-two scheme at room temperature using isothiocyano-benzyl-EDTA (ITCB-EDTA) containing isotopically enriched Palladium ions with atomic masses of 102, 104, 106, 108, and 110 Da [48]. Each run contained five patient and five control samples. A reference sample, labelled with mDOTA-Rh103, was included in each run to monitor assay performance. Assignment of patient and control samples to individual barcodes and runs is included in Supplementary Table S1B,C. After barcoding, samples were washed twice with 3 mL cell staining medium and then pooled. Fc receptor blocking was performed for 10 min at room temperature using 0.2 mg/mL Beriglobin (CSL Behring, King of Prussia, PA, USA), followed by a washing step with 2 mL cell staining medium. Cells were then incubated for 15 min in heparin–sodium (Ratiopharm, Ulm, Germany) at a final concentration of 100 U/mL in cell staining medium (heparin-CSM) at room temperature, as described before [49]. Cell-surface staining was performed for 30 min at room temperature in the presence of heparin and stopped by addition of 2 mL cell staining medium. Antibodies are listed in Supplementary Table S1A. Cells were pelleted, and washed once more with 2 mL PBS before fixation with 4% paraformaldehyde solution (Electron Microscopy Sciences, Hatfield, PA, USA) for 10 min at room temperature. After fixation, cells were centrifuged and gently resuspended in 1.6 mL −20 °C cold methanol (Carl Roth, Karlsruhe, Germany). Samples were stored at −80 °C overnight. The following day, cells were washed twice with 4 mL cell staining medium, followed by another 15 min- blocking step with heparin-CSM at room temperature. Antibodies directed against intracellular antigens (Supplementary Table S1A) were added directly to the cell suspension and incubated for 60 min at room temperature. Cells were then washed once with 2 mL cell staining medium and PBS before fixing the cells with 2% paraformaldehyde for 10 min at room temperature. Next, the samples were incubated at room temperature for 25 min in 2 mL PBS supplemented with 1:500 (*v/v*) 0.125 mM iridium-based DNA intercalator (Fluidigm, South San Francisco, CA, USA). Cells were then washed twice with 2 mL cell staining medium and twice with 2 mL deionized water, before filtering samples through a 30 µm cell strainer (Corning, Corning, NY, USA). Finally, the sample was resuspended at 7.5 × 105 cells/mL, and 10% (*v/v*) EQ Four Element Calibration Beads (Fluidigm, South San Francisco, CA, USA) were added to the sample before acquisition. Cells were acquired on a mass cytometer (Helios, Fluidigm, South San Francisco, CA, USA) at a rate of 250 to 350 events per second. Raw mass cytometry data were converted to Flow Cytometry Standard 3.0 files during acquisition. Data files were normalized using the Helios software version 6.7.1014 based on EQ Four Element Calibration Beads passport P13H2302.

Prior to analysis, all cytometric channels except time, event length, and the Gaussian parameters were arcsinh transformed with a scale argument of 5. Intact, nucleated cells events were identified by exclusion of beads according to their 140Ce signal, and by gating on events stained by iridium intercalator and CD45 antibody. Cells with an event length of >30 were excluded to minimize doublets. Cell samples were debarcoded by Boolean gating [50,51]. Compensation of signal spillover was performed as described [52,53]. On average, 190.921 cells were included from each donor (range, 131.628–200.000 cells), yielding 7.64 × 106 total cells, which were used for downstream analyses.

### 4.4. PBMC Isolation

For flow cytometry, peripheral blood mononuclear cells (PBMCs) were isolated and stained as previously described [54]. In short, 35 mL of a 1:1 (*v/v*) mix of heparinized blood and phosphate-buffered saline supplemented with 0.2% bovine serum albumin (PBS/BSA) were layered onto 15 mL of Ficoll-Paque PLUS (GE Healthcare, Chicago, IL, USA) in 50 mL centrifuge tubes. After centrifugation (20 min at room temperature), PBMCs were isolated and washed twice in cold PBS/BSA, and were kept on ice for further use.

### 4.5. Flow Cytometry

Approximately 0.5 × 10⁶ cells were stained in 100 µL PBS for 15 min with the antibodies indicated in Supplementary Table S2 as well as the Fixable Viability Dye eFluor™ 780 (ThermoFisher, Waltham, MA, USA) to stain dead cells. The samples were acquired on a FACSCanto flow cytometer (BD Biosciences, Franklin Lakes, NJ, USA). All cytometry experiments were performed according to published guidelines [55]. In order to ensure comparability of fluorescence intensities across measurements, daily bead calibration was performed and samples from patients and controls were preferentially measured on the same day. Cytometry data were analyzed using FlowJo v10.6.2 (FlowJo, LLC, Ashland, OR, USA) for Mac.

### 4.6. Data Analysis

Statistical analyses and visualizations were created using Prism v9.0.0 (GraphPad, San Diego, CA, USA). For comparisons of CD38 expression between healthy controls and SLE patients, the Mann–Whitney test was used. Correlation analysis of CD38 expression between different cell types or subsets was performed using Spearman correlation. Multidimensional scaling (mds) was performed using base R [56], as well as the tidyverse [57] and ggplot2 [58] packages. Dimension reduction by t-SNE was performed using the opt-SNE [19] implementation in OMIQ.ai (Santa Clara, CA, USA) with the default settings. The markers used for opt-SNE calculations are indicated in Supplementary Table S1A. On average, 99% of all events were assigned to one of the major leukocyte lineages shown in Figure 1A, according to their expression of lineage-defining markers (Supplementary Figure S1). The remaining events (not annotated in Figure 1A) were excluded from further analysis. On average, 4939 B cells (range 270–13.608, in total 1.97 × 10^5^ cells) and 37.709 T cells per donor (1.147–58.041, in total 1.50 × 10^6^ cells) were used for analyses shown in Figure 2 and Figure 3. FlowSOM clustering [59,60] was performed in omiq.ai using the markers listed in Supplementary Table S1, using a 10 × 10 grid, Euclidean distance, and consensus metaclustering. The final number of metaclusters was set to 10 for B and 15 for T cell analyses.

## Figures and Tables

**Table 1:** Patient characteristics.

	Mass Cytometry Cohort			Flow Cytometry Cohort		
	Healthy Controls	SLE Patients	*p* Value	Healthy Controls	SLE Patients	*p* Value
Number	20	20		19	36	
Age (median, IQR)	39 (30–46)	39 (30–47)	0.732	28 (24–30)	40 (31–46)	0.001
Sex (*n*% female)	18 (90.0%)	18 (90.0%)	1.00	11 (57.9%)	33 (91.7%)	0.003
SLEDAI-2K (median, range)	–	8 (2–14)		–	4.5 (0–14)	
Clinically active SLE (clinical SLEDAI-2K > 0)	–	17 (85.0%)		–	24 (66.7%)	
Serologically active SLE (serological SLEDAI-2K > 0)	–	18 (90%)		–	29 (80.6%)	
Prednisolone Dose (mg/d; median, range)	–	5.0 (0–20.0)		–	5.0 (0–25.0)	
Other Immunosuppressive Medication (*n*)	–	HCQ: 17 (85.0%) AZA: 4 (20.0%) MTX: 3 (15.0%) BEL: 3 (15.0%) MMF: 1 (5.0%)		–	HCQ: 24 (66.7%) MMF: 9 (25.0%) AZA: 9 (25.0%) CsA: 4 (11.1%) BEL: 4 (11.1%) RTX: 3 (8.3%) UST: 1 (2.8%)	

Abbreviations: Hydroxychloroquine (HCQ), Azathioprine (AZA), Methotrexate (MTX), Belimumab (BEL), Mycophenolate Mofetil (MMF), Ciclosporin A (CsA), Rituximab (RTX), Ustekinumab (UST). Statistical analysis comparing differences in age was performed using the Mann–Whitney test, sex differences with the chi-square test.

## Data Availability

The data from this study are available from the corresponding author upon request.

## Supplementary Materials

The following are available online at https://www.mdpi.com/1422-0067/22/5/2424/s1: Supplementary Figure S1: T-SNE map of mass cytometry data from Figure 1A, colored by the expression of lineage-defining cell-surface markers which were used to annotate the major immune cell subsets., Supplementary Figure S2: Analysis of coexpression of CD38 and myeloid markers in innate immune cells and CD38 expression peripheral blood monocytes in the context of SIGLEC-1 expression. Supplementary Figure S3: Analysis of B cell subsets and PB/PC for their CD38 expression in patients with SLE. Supplementary Figure S4: Analysis of T cell subsets for their CD38 expression in patients with SLE. Supplementary Figure S5: Differential correlation of CD38 expression among immune cell subsets between SLE and controls. Supplementary Table S1: Mass cytometry antibodies and details, Supplementary Table S2: Flow Cytometry antibodies.

## References

[1] Tsokos G.C. (2011). Systemic Lupus Erythematosus. N. Engl. J. Med..

[2] Lande R., Ganguly D., Facchinetti V., Frasca L., Conrad C., Gregorio J., Meller S., Chamilos G., Sebasigari R., Riccieri V. (2011). Neutrophils Activate Plasmacytoid Dendritic Cells by Releasing Self-DNA-Peptide Complexes in Systemic Lupus Erythematosus. Sci. Transl. Med..

[3] Biesen R., Demir C., Barkhudarova F., Grün J.R., Steinbrich-Zöllner M., Backhaus M., Häupl T., Rudwaleit M., Riemekasten G., Radbruch A. (2008). Sialic acid-binding Ig-like lectin 1 expression in inflammatory and resident monocytes is a potential biomarker for monitoring disease activity and success of therapy in systemic lupus erythematosus. Arthritis Rheum..

[4] Hiepe F., Radbruch A. (2016). Plasma cells as an innovative target in autoimmune disease with renal manifestations. Nat. Rev. Nephrol..

[5] Hogan K.A., Chini C.C.S., Chini E.N. (2019). The Multi-faceted Ecto-enzyme CD38: Roles in Immunomodulation, Cancer, Aging, and Metabolic Diseases. Front. Immunol..

[6] Deaglio S., Morra M., Mallone R., Ausiello C.M., Prager E., Garbarino G., Dianzani U., Stockinger H., Malavasi F. (1998). Human CD38 (ADP-ribosyl cyclase) is a counter-receptor of CD31, an Ig superfamily member. J. Immunol..

[7] Partida-Sánchez S., Cockayne D.A., Monard S., Jacobson E.L., Oppenheimer N., Garvy B., Kusser K., Goodrich S., Howard M., Harmsen A. (2001). Cyclic ADP-ribose production by CD38 regulates intracellular calcium release, extracellular calcium influx and chemotaxis in neutrophils and is required for bacterial clearance in vivo. Nat. Med..

[8] Partida-Sánchez S., Goodrich S., Kusser K., Oppenheimer N., Randall T.D., Lund F.E. (2004). Regulation of Dendritic Cell Traf-ficking by the ADP-Ribosyl Cyclase CD38. Immunity.

[9] Katsuyama E., Suarez-Fueyo A., Bradley S.J., Mizui M., Marin A.V., Mulki L., Krishfield S., Malavasi F., Yoon J., Sui S.J.H. (2020). The CD38/NAD/SIRTUIN1/EZH2 Axis Mitigates Cytotoxic CD8 T Cell Function and Identifies Patients with SLE Prone to Infections. Cell Rep..

[10] Pavón E.J., Zumaquero E., Rosal-Vela A., Khoo K.-M., Cerezo-Wallis D., García-Rodríguez S., Carrascal M., Abián J., Graeff R., Callejas-Rubio J.-L. (2013). Increased CD38 expression in T cells and circulating anti-CD38 IgG autoantibodies differentially correlate with distinct cytokine profiles and disease activity in systemic lupus erythematosus patients. Cytokine.

[11] Ostendorf L., Burns M., Durek P., Heinz G.A., Heinrich F., Garantziotis P., Enghard P., Richter U., Biesen R., Schneider U. (2020). Targeting CD38 with Daratumumab in Refractory Systemic Lupus Erythematosus. New Engl. J. Med..

[12] Perry D.J., Titov A.A., Sobel E.S., Brusko T.M., Morel L. (2020). Immunophenotyping reveals distinct subgroups of lupus patients based on their activated T cell subsets. Clin. Immunol..

[13] Amici S.A., Young N.A., Narvaez-Miranda J., Jablonski K.A., Arcos J., Rosas L., Papenfuss T.L., Torrelles J.B., Jarjour W.N., Guerau-De-Arellano M. (2018). CD38 Is Robustly Induced in Human Macrophages and Monocytes in Inflammatory Conditions. Front. Immunol..

[14] Burlock B., Richardson G., García-Rodríguez S., Guerrero S., Zubiaur M., Sancho J. (2018). The Role of CD38 on the Function of Regulatory B Cells in a Murine Model of Lupus. Int. J. Mol. Sci..

[15] Cole S., Walsh A., Yin X., Wechalekar M.D., Smith M.D., Proudman S.M., Veale D.J., Fearon U., Pitzalis C., Humby F. (2018). Integrative analysis reveals CD38 as a therapeutic target for plasma cell-rich pre-disease and established rheumatoid arthritis and systemic lupus erythematosus. Arthritis Res..

[16] Scheibe F., Ostendorf L., Reincke S.M., Prüss H., Von Brünneck A.-C., Köhnlein M., Alexander T., Meisel C., Meisel A. (2019). Daratumumab treatment for therapy-refractory anti-CASPR2 encephalitis. J. Neurol..

[17] Ratuszny D., Skripuletz T., Wegner F., Groß M., Falk C., Jacobs R., Ruschulte H., Stangel M., Sühs K.-W. (2020). Case Report: Daratumumab in a Patient With Severe Refractory Anti-NMDA Receptor Encephalitis. Front. Neurol..

[18] Schuetz C., Hoenig M., Moshous D., Weinstock C., Castelle M., Bendavid M., Shimano K., Tolbert V., Schulz A.S., Dvorak C.C. (2018). Daratumumab in life-threatening autoimmune hemolytic anemia following hematopoietic stem cell transplantation. Blood Adv..

[19] Belkina A.C., Ciccolella C.O., Anno R., Halpert R., Spidlen J., Snyder-Cappione J.E. (2019). Automated optimized parameters for T-distributed stochastic neighbor embedding improve visualization and analysis of large datasets. Nat. Commun..

[20] Rose T., Grützkau A., Hirseland H., Huscher D., Dähnrich C., Dzionek A., Ozimkowski T., Schlumberger W., Enghard P., Radbruch A. (2012). IFNα and its response proteins, IP-10 and SIGLEC-1, are biomarkers of disease activity in systemic lupus erythematosus. Ann. Rheum. Dis..

[21] Odendahl M., Mei H., Hoyer B.F., Jacobi A.M., Hansen A., Muehlinghaus G., Berek C., Hiepe F., Manz R., Radbruch A. (2005). Generation of migratory antigen-specific plasma blasts and mobilization of resident plasma cells in a secondary immune response. Blood.

[22] Wang S., Wang J., Kumar V., Karnell J.L., Naiman B., Gross P.S., Rahman S., Zerrouki K., Hanna R., Autoimmunity Molecular Medicine Team (2018). IL-21 drives expansion and plasma cell differentiation of autoreactive CD11chiT-bet+ B cells in SLE. Nat. Commun..

[23] Karnell J.L., Kumar V., Wang J., Wang S., Voynova E., Ettinger R. (2017). Role of CD11c + T-bet + B cells in human health and disease. Cell. Immunol..

[24] Jacobi A.M., Mei H., Hoyer B.F., Mumtaz I.M., Thiele K., Radbruch A., Burmester G.-R., Hiepe F., Dörner T. (2009). HLA-DRhigh/CD27high plasmablasts indicate active disease in patients with systemic lupus erythematosus. Ann. Rheum. Dis..

[25] Mei H.E., Hahne S., Redlin A., Hoyer B.F., Wu K., Baganz L., Lisney A.R., Alexander T., Rudolph B., Dörner T. (2017). Plasmablasts With a Mucosal Phenotype Contribute to Plasmacytosis in Systemic Lupus Erythematosus. Arthritis Rheumatol..

[26] Odendahl M., Jacobi A., Hansen A., Feist E., Hiepe F., Burmester G.R., Lipsky P.E., Radbruch A., Dörner T. (2000). Disturbed Peripheral B Lymphocyte Homeostasis in Systemic Lupus Erythematosus. J. Immunol..

[27] Grzywacz B., Kataria N., Verneris M.R. (2007). CD56(dim)CD16(+) NK cells downregulate CD16 following target cell induced activation of matrix metalloproteinases. Leuk..

[28] Sandoval-Montes C., Santos-Argumedo L. (2004). CD38 is expressed selectively during the activation of a subset of mature T cells with reduced proliferation but improved potential to produce cytokines. J. Leukoc. Biol..

[29] Mihara K., Yoshida T., Ishida S., Takei Y., Kitanaka A., Shimoda K., Morishita K., Takihara Y., Ichinohe T. (2016). All-trans retinoic acid and interferon-α increase CD38 expression on adult T-cell leukemia cells and sensitize them to T cells bearing anti-CD38 chimeric antigen receptors. Blood Cancer J..

[30] Palanichamy A., Barnard J., Zheng B., Owen T., Quach T., Wei C., Looney R.J., Sanz I., Anolik J.H. (2009). Novel Human Transitional B Cell Populations Revealed by B Cell Depletion Therapy. J. Immunol..

[31] Sims G.P., Ettinger R., Shirota Y., Yarboro C.H., Illei G.G., Lipsky P.E. (2005). Identification and characterization of circulating human transitional B cells. Blood.

[32] Tenca C., Merlo A., Zarcone D., Saverino D., Bruno S., De Santanna A., Ramarli D., Fabbi M., Pesce C., Deaglio S. (2003). Death of T cell precursors in the human thymus: A role for CD38. Int. Immunol..

[33] Bendall S.C., Davis K.L., Amir E.-A.D., Tadmor M.D., Simonds E.F., Chen T.J., Shenfeld D.K., Nolan G.P., Pe’Er D. (2014). Single-Cell Trajectory Detection Uncovers Progression and Regulatory Coordination in Human B Cell Development. Cell.

[34] Blair P.A., Noreña L.Y., Flores-Borja F., Rawlings D.J., Isenberg D.A., Ehrenstein M.R., Mauri C. (2010). CD19+CD24hiCD38hi B Cells Exhibit Regulatory Capacity in Healthy Individuals but Are Functionally Impaired in Systemic Lupus Erythematosus Patients. Immunity.

[35] Li R., Rezk A., Miyazaki Y., Hilgenberg E., Touil H., Shen P., Moore C.S., Michel L., Althekair F., Rajasekharan S. (2015). Proinflammatory GM-CSF–producing B cells in multiple sclerosis and B cell depletion therapy. Sci. Transl. Med..

[36] Deterre P., Berthelier V., Bauvois B., Dalloul A., Schuber F., Lund F. (2000). CD38 in T-and B-cell functions. Human CD38 and Related Molecules.

[37] Patton D.T., Wilson M.D., Rowan W.C., Soond D.R., Okkenhaug K. (2011). The PI3K p110δ Regulates Expression of CD38 on Regulatory T Cells. PLoS ONE.

[38] Quách T.D., Rodríguez-Zhurbenko N., Hopkins T.J., Guo X., Hernández A.M., Li W., Rothstein T.L. (2016). Distinctions among Circulating Antibody-Secreting Cell Populations, Including B-1 Cells, in Human Adult Peripheral Blood. J. Immunol..

[39] Griffin D.O., Holodick N.E., Rothstein T.L. (2011). Human B1 cells in umbilical cord and adult peripheral blood express the novel phenotype CD20+CD27+CD43+CD70−. J. Exp. Med..

[40] Griffin D.O., Rothstein T.L. (2011). A small CD11b+ human B1 cell subpopulation stimulates T cells and is expanded in lupus. J. Exp. Med..

[41] Musso T., Deaglio S., Franco L., Calosso L., Badolato R., Garbarino G., Dianzani U., Malavasi F. (2001). CD38 expression and functional activities are up-regulated by IFN-gamma on human monocytes and monocytic cell lines. J. Leukoc. Biol..

[42] Kang B., Tirumurugaan K.G., Deshpande D.A., Amrani Y., Panettieri R.A., Walseth T.F., Kannan M.S. (2006). Transcriptional regulation of CD38 expression by tumor necrosis factor-α in human airway smooth muscle cells: Role of NF-κB and sensitivity to glucocor-ticoids. FASEB J..

[43] Rahil Z., Leylek R., Schürch C.M., Chen H., Bjornson-Hooper Z., Christensen S.R., Gherardini P.F., Bhate S.S., Spitzer M.H., Fragiadakis G.K. (2020). Landscape of coordinated immune responses to H1N1 challenge in humans. J. Clin. Investig..

[44] Park Y.-W., Kee S.-J., Cho Y.-N., Lee E.-H., Lee H.-Y., Kim E.-M., Shin M.-H., Park J.-J., Kim T.-J., Lee S.-S. (2009). Impaired differentiation and cytotoxicity of natural killer cells in systemic lupus erythematosus. Arthritis Rheum..

[45] Schepis D., Gunnarsson I., Eloranta M.L., Lampa J., Jacobson S.H., Kärre K., Berg L. (2009). Increased proportion of CD56bright natural killer cells in active and inactive systemic lupus erythematosus. Immunology.

[46] Aringer M., Costenbader K., Daikh D., Brinks R., Mosca M., Ramsey-Goldman R., Smolen J.S., Wofsy D., Boumpas D.T., Kamen D.L. (2019). 2019 European League Against Rheumatism/American College of Rheumatology Classification Criteria for Systemic Lupus Erythematosus. Arthritis Rheumatol..

[47] Gladman D.D., Ibañez M., Urowitz M.B. (2002). Systemic lupus erythematosus disease activity index 2000. J. Rheumatol..

[48] Zunder E.R., Finck R., Behbehani G.K., Amir E.D., Krishnaswamy S., Gonzalez V.D., Lorang C.G., Bjornson Z., Spitzer M.H., Bodenmiller B. (2015). Palladium-based mass tag cell barcoding with a doublet-filtering scheme and single-cell deconvolution algorithm. Nat. Protoc..

[49] Rahman A.H., Tordesillas L., Berin M.C. (2016). Heparin reduces nonspecific eosinophil staining artifacts in mass cytometry experiments. Cytom. Part A.

[50] Mei H.E., Leipold M.D., Schulz A.R., Chester C., Maecker H.T. (2015). Barcoding of Live Human Peripheral Blood Mononuclear Cells for Multiplexed Mass Cytometry. J. Immunol..

[51] Olsen L.R., Leipold M.D., Pedersen C.B., Maecker H.T. (2019). The anatomy of single cell mass cytometry data. Cytom. Part A.

[52] Chevrier S., Crowell H.L., Zanotelli V.R., Engler S., Robinson M.D., Bodenmiller B. (2018). Compensation of Signal Spillover in Suspension and Imaging Mass Cytometry. Cell Syst..

[53] Budzinski L., Schulz A.R., Baumgart S., Burns T., Rose T., Hirseland H., Mei H.E. (2019). Osmium-Labeled Microspheres for Bead-Based Assays in Mass Cytometry. J. Immunol..

[54] Ostendorf L., Mothes R., Van Koppen S., Lindquist R.L., Bellmann-Strobl J., Asseyer S., Ruprecht K., Alexander T., Niesner R.A., Hauser A.E. (2019). Low-Density Granulocytes Are a Novel Immunopathological Feature in Both Multiple Sclerosis and Neuromyelitis Optica Spectrum Disorder. Front. Immunol..

[55] Cossarizza A., Chang H., Radbruch A., Acs A., Adam D., Adam-Klages S., Agace W.W., Aghaeepour N., Akdis M., Allez M. (2019). Guidelines for the use of flow cytometry and cell sorting in immunological studies (second edition). Eur. J. Immunol..

[56] R Core Team (2018). R: A Language and Environment for Statistical Computing.

[57] Wickham H., Averick M., Bryan J., Chang W., McGowan L., François R., Grolemund G., Hayes A., Henry L., Hester J. (2019). Welcome to the Tidyverse. J. Open Source Softw..

[58] Wickham H. (2009). Ggplot2: Elegant Graphics for Data Analysis.

[59] Van Gassen S., Callebaut B., Van Helden M.J., Lambrecht B.N., Demeester P., Dhaene T., Saeys Y. (2015). FlowSOM: Using self-organizing maps for visualization and interpretation of cytometry data. Cytom. Part A.

[60] Nowicka M., Krieg C., Crowell H.L., Weber L.M., Hartmann F.J., Guglietta S., Becher B., Levesque M.P., Robinson R.B. (2019). CyTOF workflow: Differential discovery in high-throughput high-dimensional cytometry datasets. F1000Research.

