# Supplementary material for: Dysregulated CD38 Expression on Peripheral Blood Immune Cell Subsets in SLE
Source: Int J Mol Sci. 2021 Feb 28;22(5):2424. doi: 10.3390/ijms22052424 (PMC7957821; doi:10.3390/ijms22052424)
Supplement: Supplementary file 1 [file ijms-22-02424-s001.zip › ijms-1103308-supplementary.docx]

**Supplementary Materials**


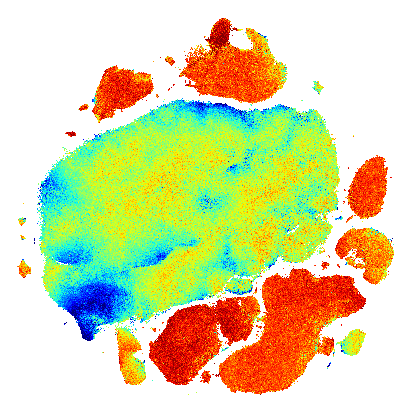

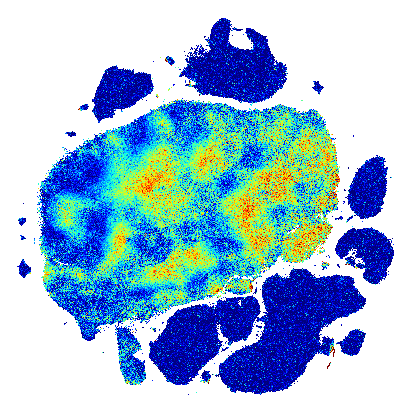

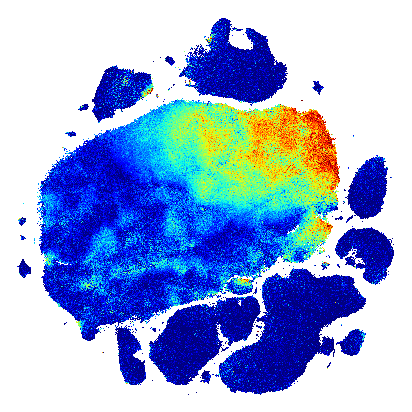

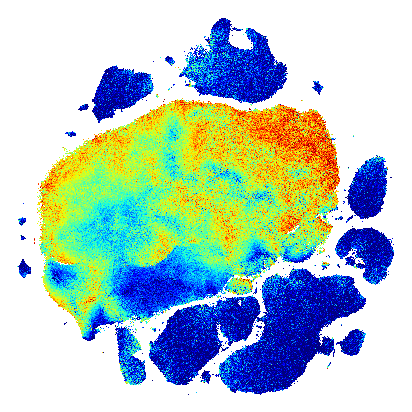

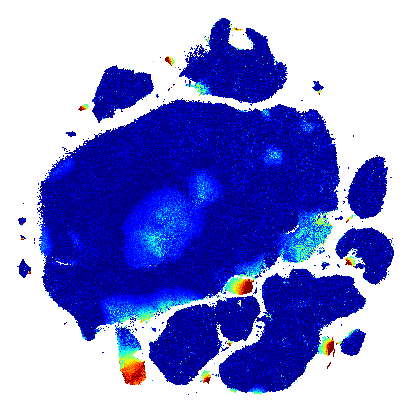

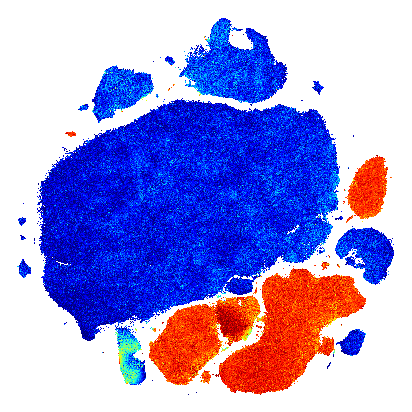

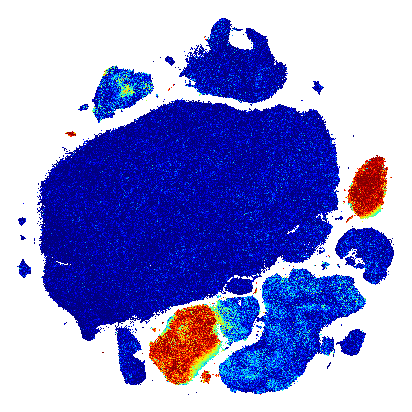

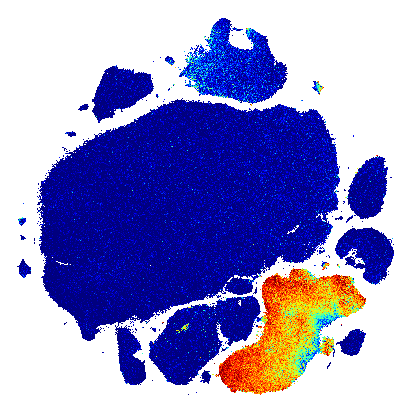

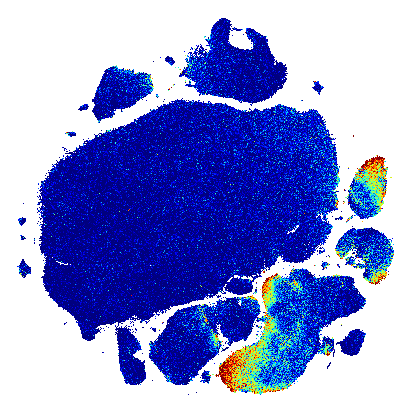

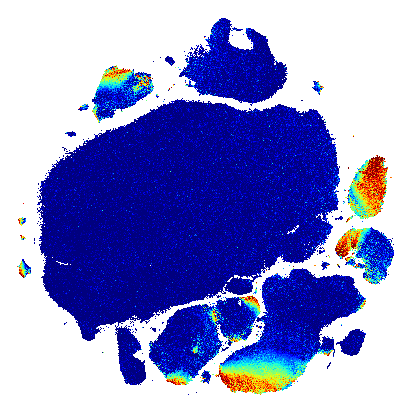

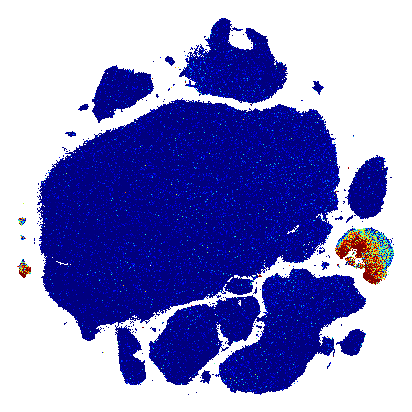

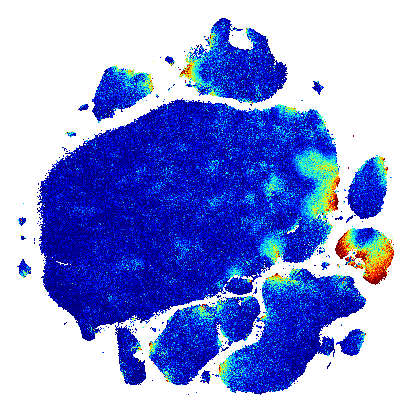

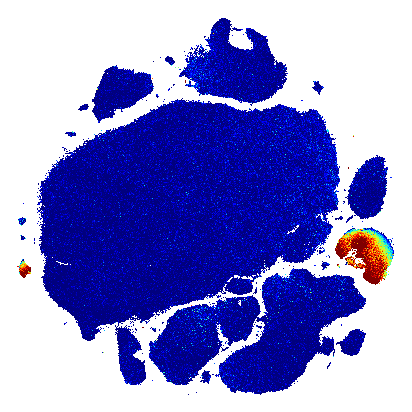

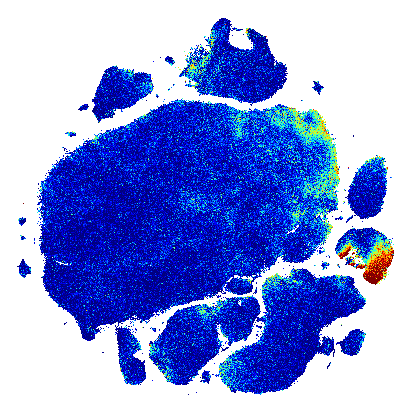

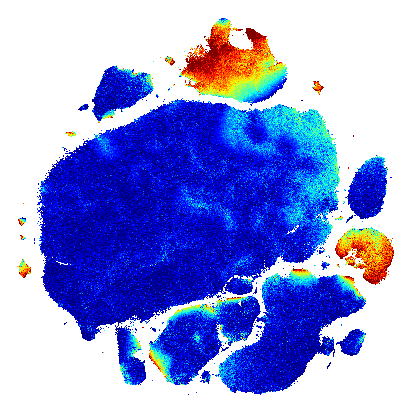


**tSNE 2**

**tSNE 1**

**CD45**

**CD66b**

**CD15**

**CD16**

**SIGLEC-8**

**CD3**

**CD4**

**CD8**

**CD45RA**

**CCR7**

**CD19**

**CD20**

**IgM**

**IgD**

**IgA**

**HLA-DR**

**CD14**

**CD123**

**CD11c**

**CD56**


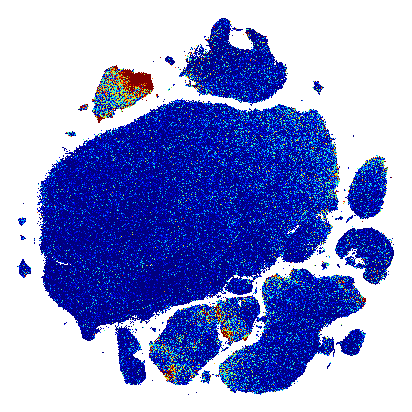

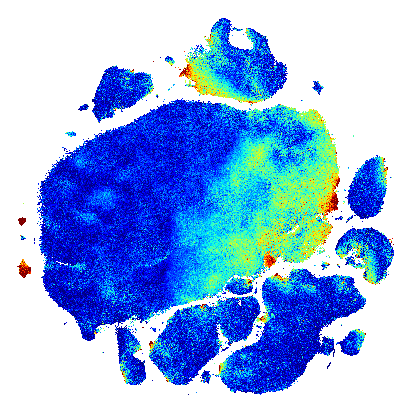

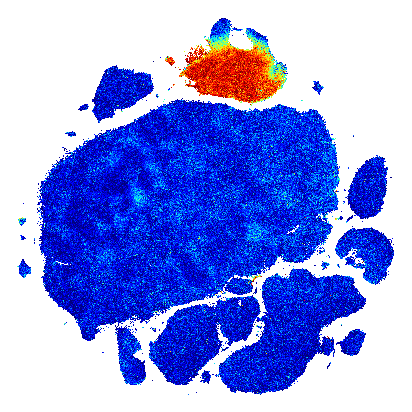

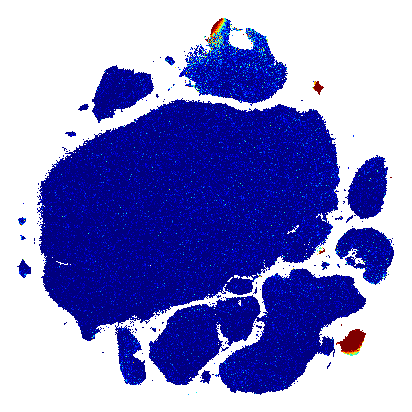

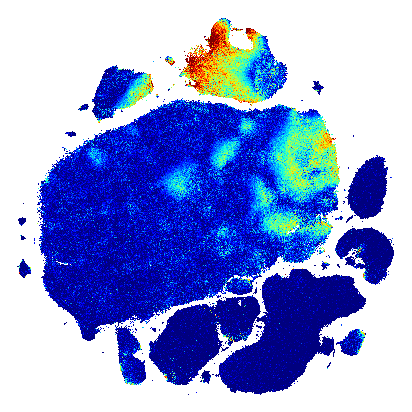


**Figure S1.** T-SNE map of mass cytometry data from Figure 1A, colored by the expression of cell-surface markers which were used to annotate the major immune cell subsets.


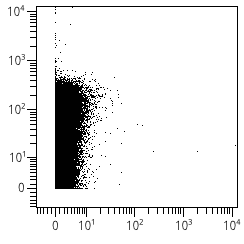

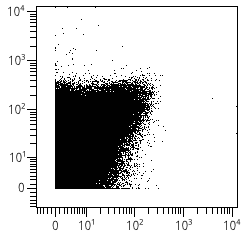


**controls**

**SLE**

**SIGLEC-1**

**CD38**

**SLE**

********


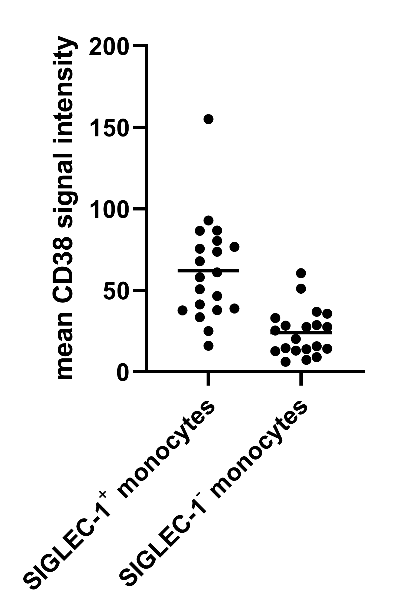


**A**

**B**

**CD38**

**HLA-DR**

**CD11c**

**Syk**

**NK cells**

**mDCs**

**pDCs**

**monocytes**

**basophils**


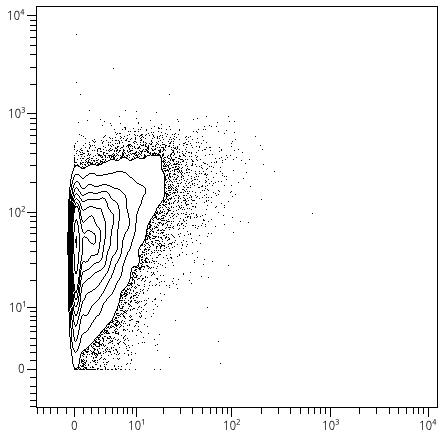

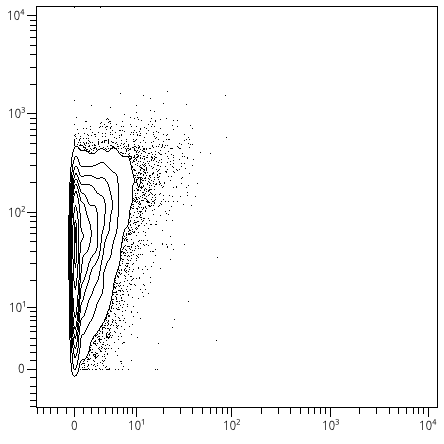

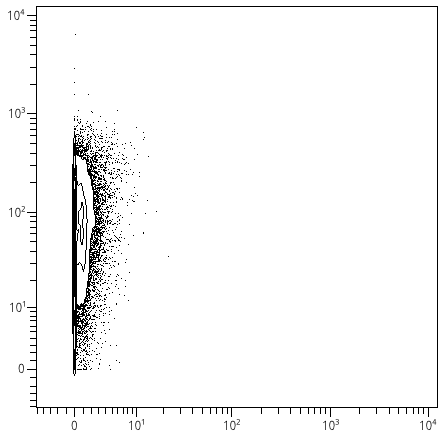

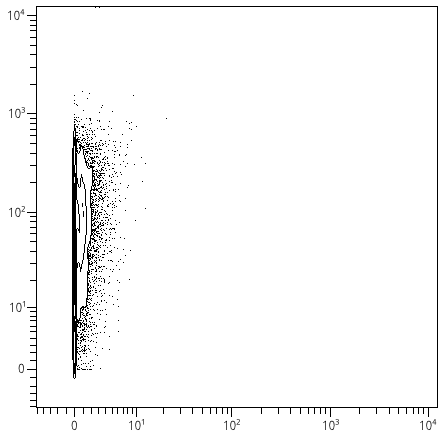

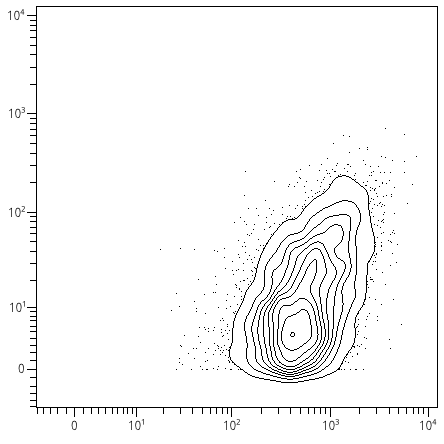

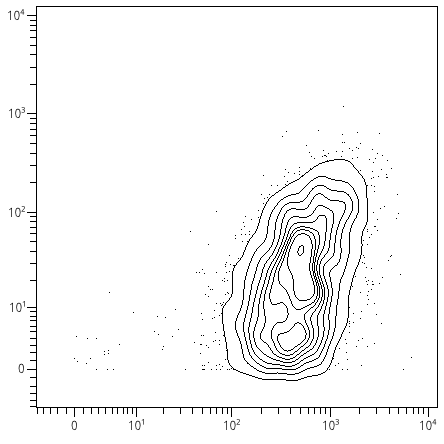

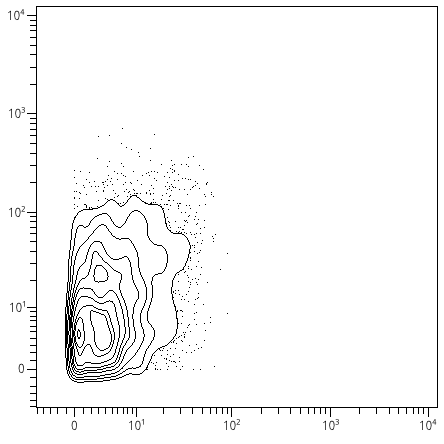

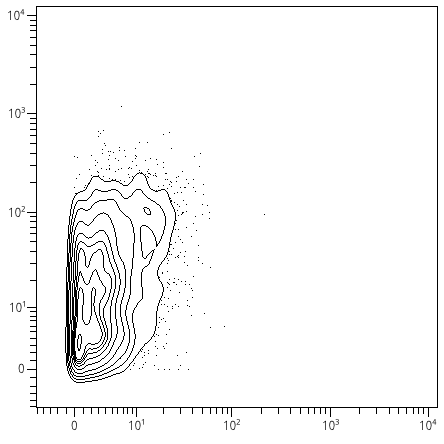

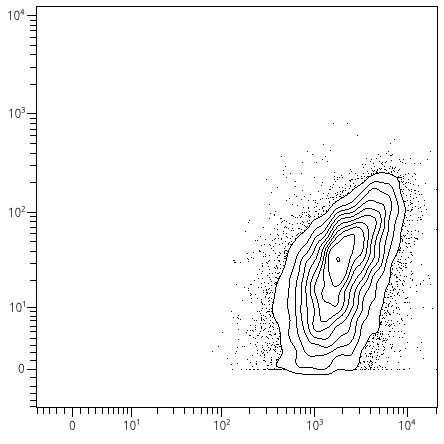

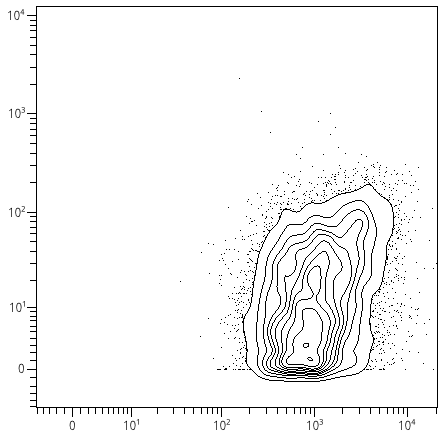

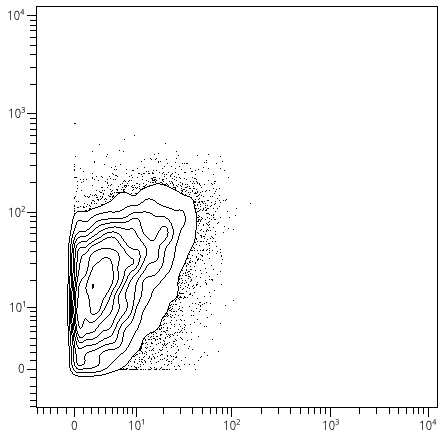

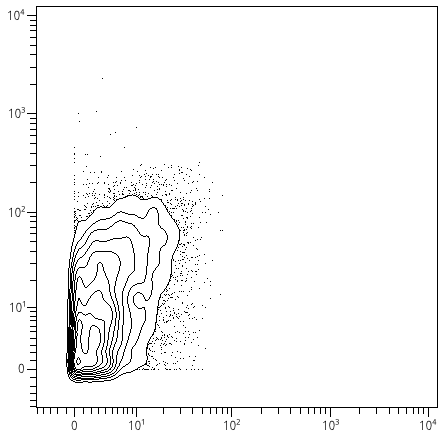

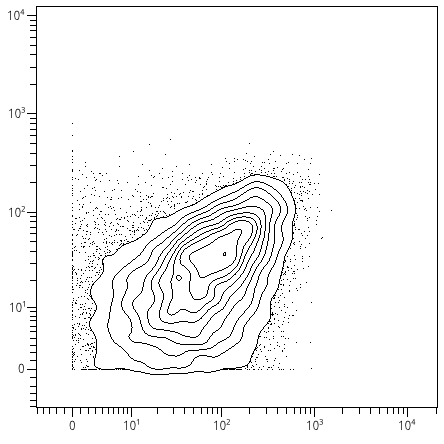

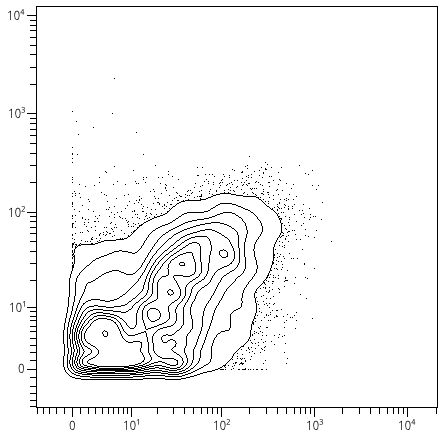

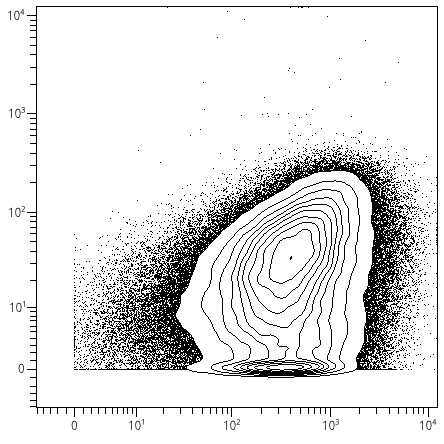

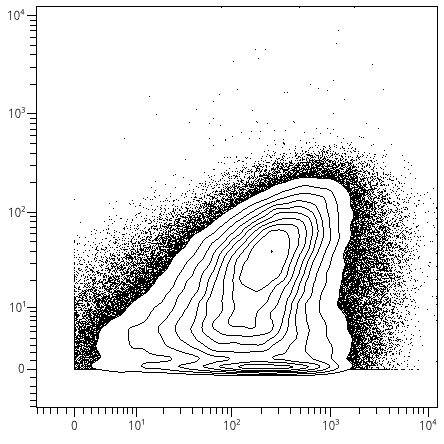

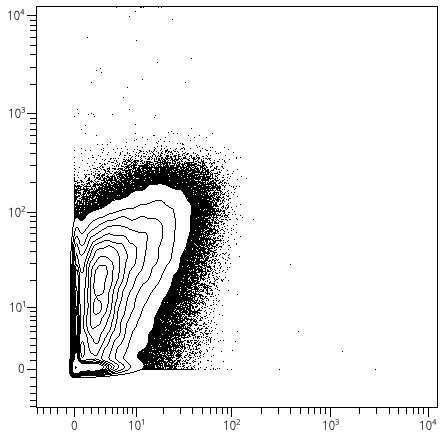

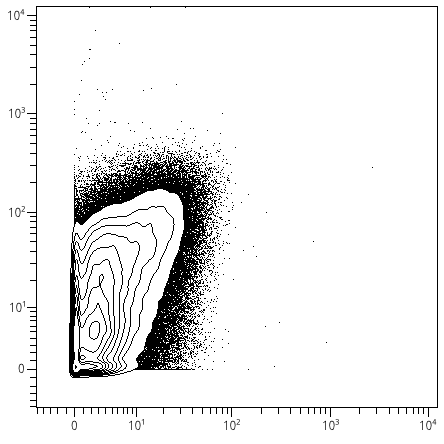

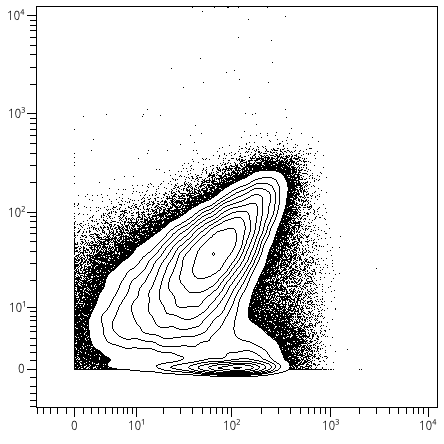

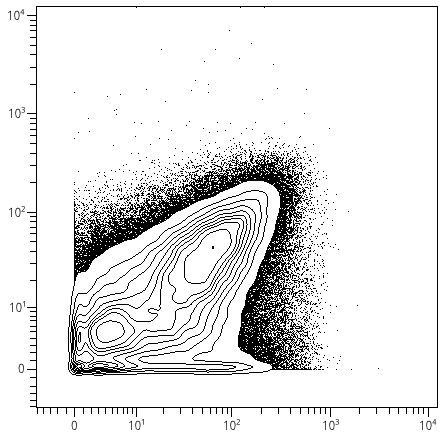

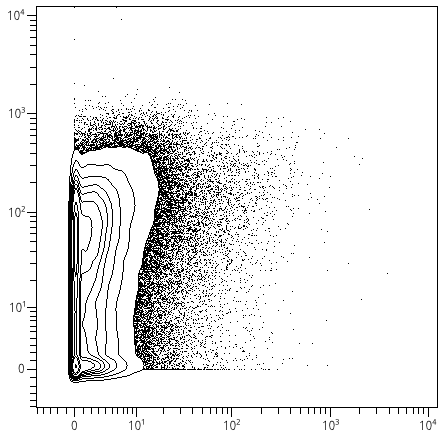

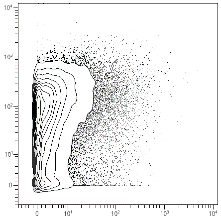

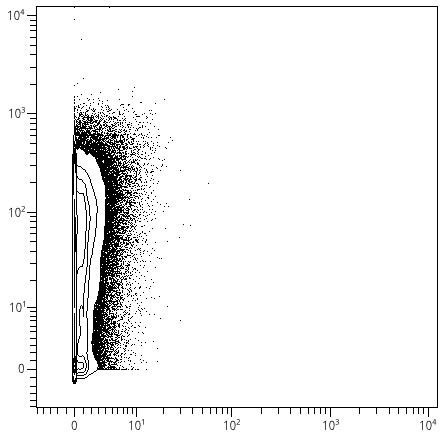

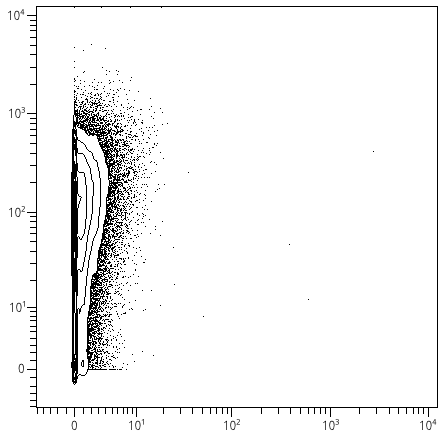

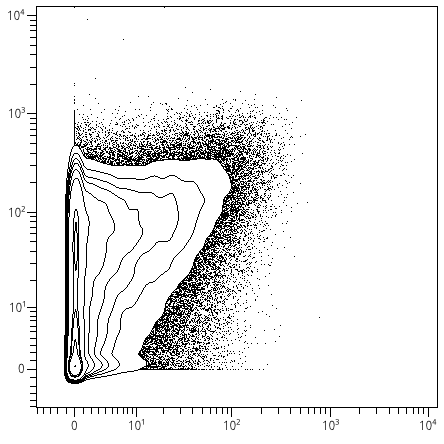

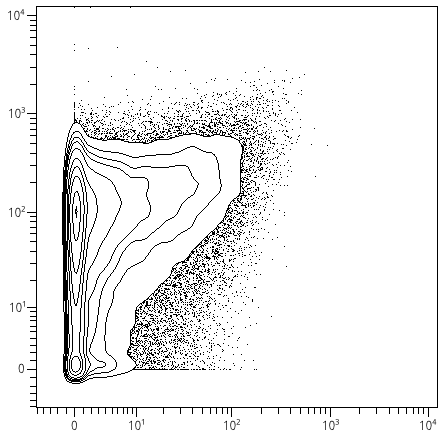


**controls**

**SLE**

**controls**

**SLE**

**controls**

**SLE**

**Figure S2.** Analysis of co-expression of CD38 and myeloid markers by innate immune cells and CD38 expression by peripheral blood monocytes in the context of SIGLEC-1 expression. (**A**) Contour plots of HLA-DR, Syk and CD11c expression on NK cells, total monocytes, myeloid (mDCs) and plasmacytoid (pDCs) dendritic cells. Concatenated data of 20 healthy controls and 20 SLE patients are shown. (**B**) Total monocytes were gated from the mass cytometry data. Dot plots show the expression of CD38 and SIGLEC-1 in SLE patients and healthy controls. Concatenated data is shown for both groups. For SLE patients, SIGLEC-1^+^ and SIGLEC-1^-/low^ monocytes were gated, and their CD38 expression was compared using the Mann-Whitney test (****, *p* < 0.0001).

**tSNE 2**

**tSNE 1**


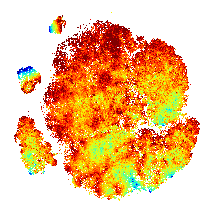

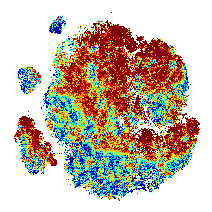

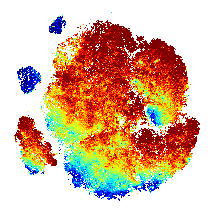

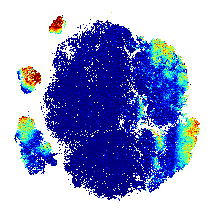


**CD19**

**CD20**

**CD27**


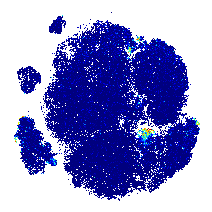

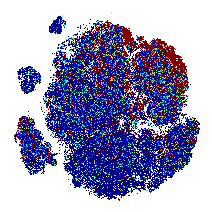

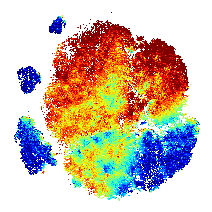

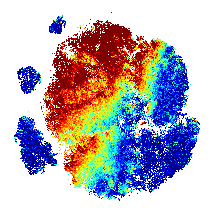

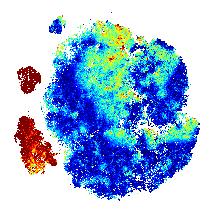

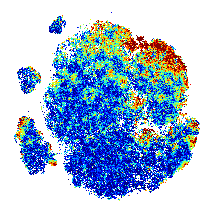


**CD1c**

**HLA-DR**

**IgM**

**IgD**

**IgA**

**CD11c**

**Syk**


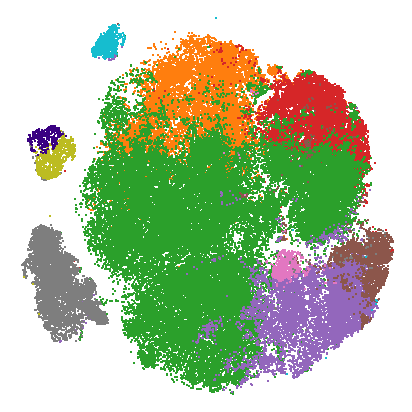


**1**

**2**

**3**

**4**

**5**

**6**

**7**

**8**

**9**

**10**

**naïve**

**naïve**

**marginal zone-like**

**CD11c^+^ B cells**

**non-IgA**

**memory B cells**

**IgD^-^ CD27^-^**

**cells**

**IgA^+^**

**B cells**

**plasmablasts**

**IgA^+^**

**plasmablasts**

**IgA^+^**

**plasma cells**

**A**

**B**


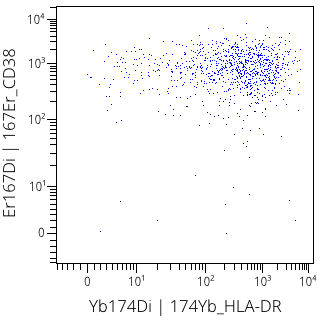

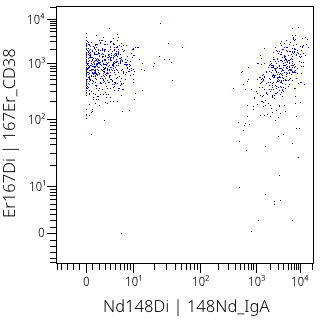


**IgA**

**CD38**

**HLA-DR**

**CD38**

**E**


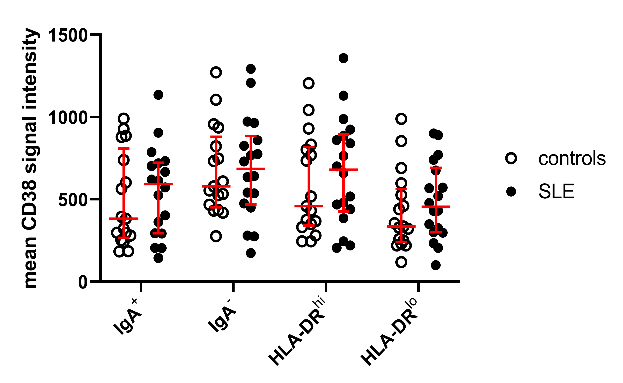


**PB/PC**

**CD45**

**CD38**


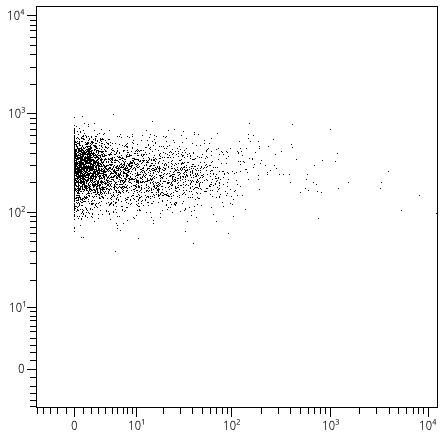

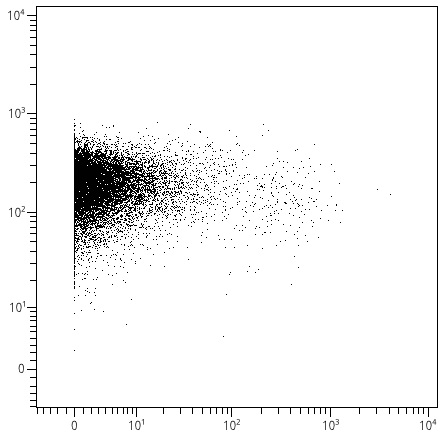

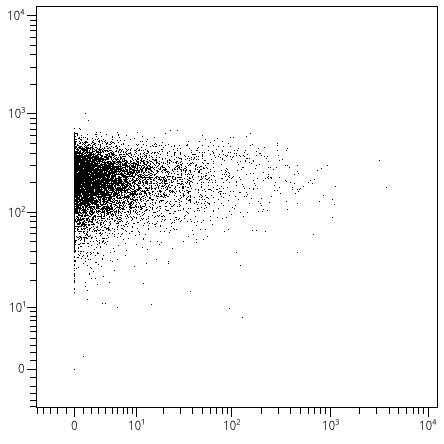

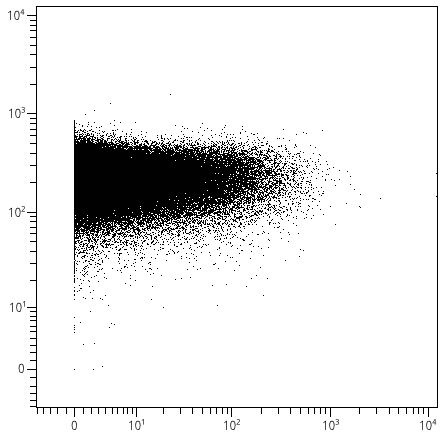


**naive B cells**

**MZ-like B cells**


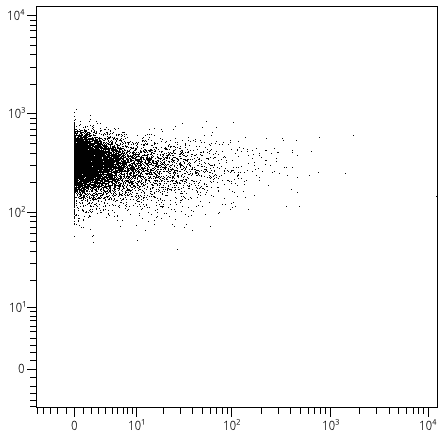


**IgD^-^ CD27^-^**

**B cells**

**non- IgA**

**memory B cells**


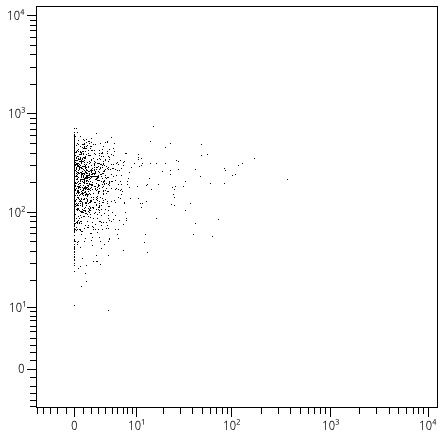


**CD11c^+^ B cells**

**IgA^+^ B cells**


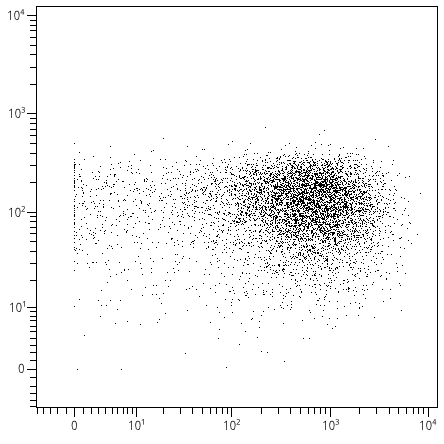


**PB/PC**

**C**

**D**


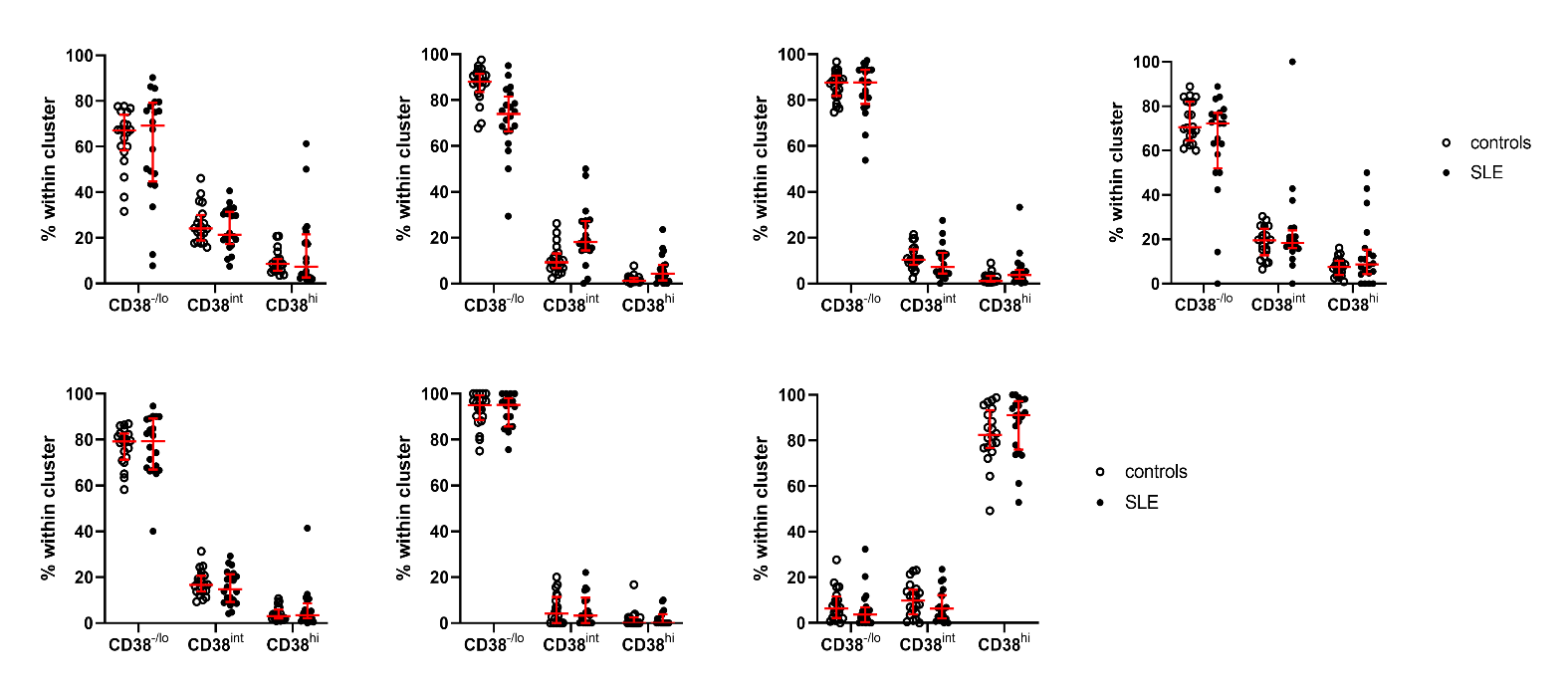


**naive B cells**

**MZ-like B cells**

**IgD^-^ CD27^-^ B cells**

**non- IgA**

**memory B cells**

**CD11c^+^ B cells**

**IgA^+^ B cells**

**PB/PC**

*******

******

*****

*****

**Figure S3.** Analysis of B cell subsets and PB/PC for their CD38 expression in patients with SLE This supplementary figure extends Figure 2. (**A**) t-SNE map showing 10 B cell clusters generated by FlowSOM. Clusters comprising naive B cells (c1, c3) and PB/PC (c8, c9, c10) were merged for further analyses in Figure 2. Concatenated data of 20 healthy controls are shown. (**B**) t-SNE map depicted in (**A**) colored by the expression of markers expressed by B cells. (**C**) B cell subsets including PB/PC were analyzed their CD38 expression, by delineating subsets expressing low or no CD38, intermediate levels of CD38, or high levels of CD38 . Concatenated data of 20 healthy controls and 20 SLE patients are shown (**D**) B cell subsets and PB/PC shown in (**C**) were analyzed for differences in abundance of CD38^-/low^, CD38^int^ and CD38^hi^ expressing cells between controls and SLE patients. Each dot represents the frequency of the indicated subset of one donor. Red lines indicate medians and interquartile ranges. Asterisks indicate significantly different abundances in SLE patients vs controls obtained by Mann-Whitney testing (*, *p* < 0.05; **, *p* < 0.01; ***, *p* < 0.001 ) (**E**) Subsets of PB/PC were gated according to the expression of IgA and HLA-DR (left) and analyzed for CD38 expression (right). Gating is shown for one representative healthy control. Data of 3 controls and 2 SLE patients were excluded from this analysis due to PB/PC counts of less than 7 cells in a given subset . In the summary plot, e ach dot represents the mean CD38 signal intensity (SI) of the indicated subset of one donor. Red lines indicate medians and interquartile ranges.

**B**


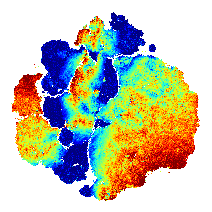

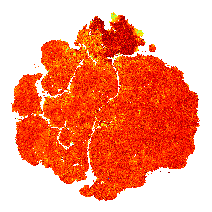

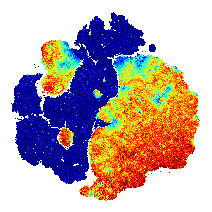

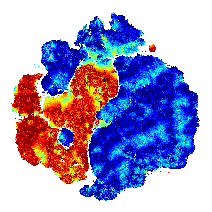

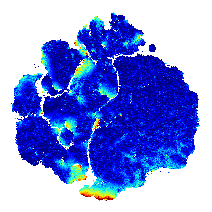

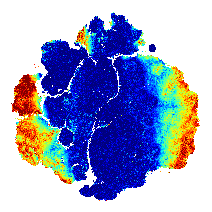

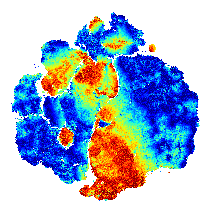

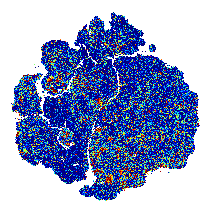

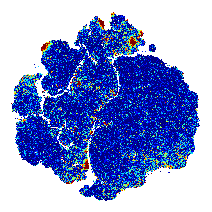

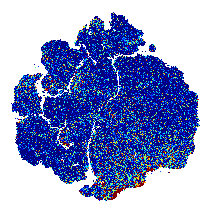

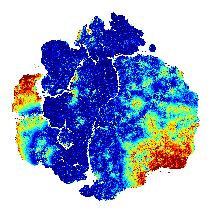

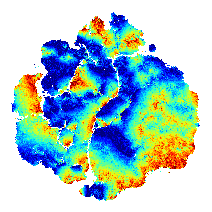


**tSNE 2**

**tSNE 1**

**CD45RO**

**CD3**

**CD4**

**CD8**

**CD45RA**

**CCR7**

**CD27**

**CD25**

**CD56**

**HLA-DR**

**CD127**

**CD7**

**A**


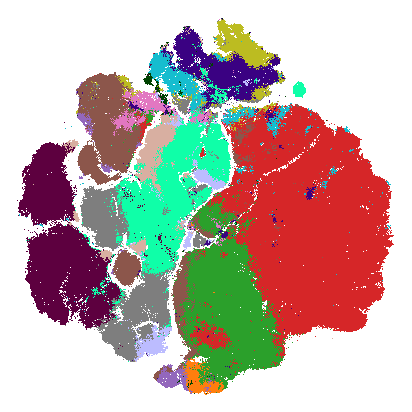


**3**

**2**

**1**

**4**

**5**

**6**

**13**

**7**

**8**

**9**

**10**

**12**

**14**

**15**

**11**

**regulatory T cells**

**CD4^+^**

**central memory**

**naive**

**CD4^+^**

**CD4^-^**

**effector memory**

**CD8^-^**

**effector memory 1**

**naive**

**CD8^-^**

**CD8^+^**

**effector memory 2**

**CD8^+^**

**central memory**

**CD4^-^ CD8^-^ T cells**

**C**

**D**

**CD4+**

**central memory**

**naive CD4^+^ T cells**

**CD4^+^**

**effector memory**

**CD45**

**CD38**

**CD8+**

**central memory**

**naive CD8^+^ T cells**

**CD8^+^**

**effector memory 1**

**CD8^+^**

**effector memory 2**


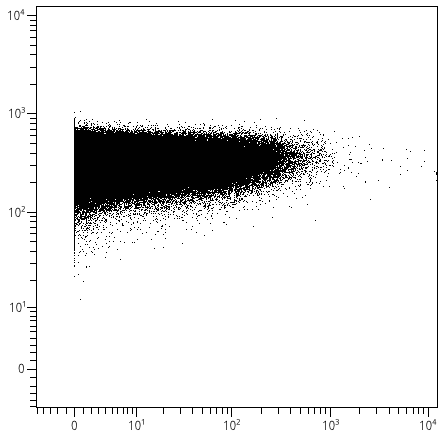

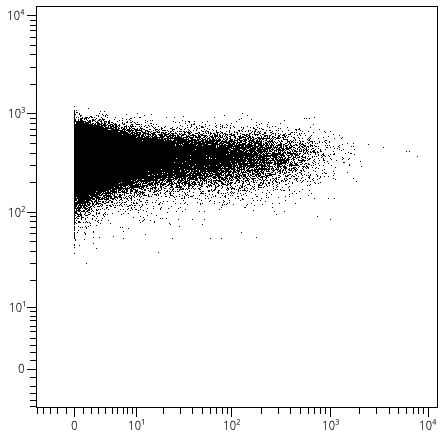

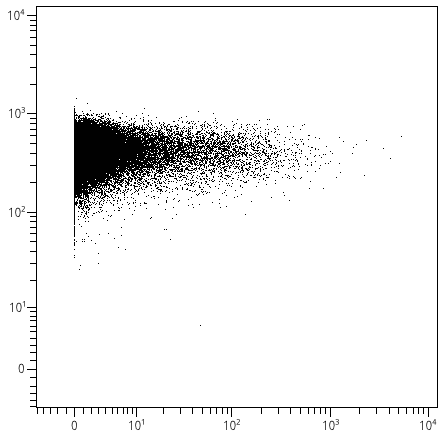

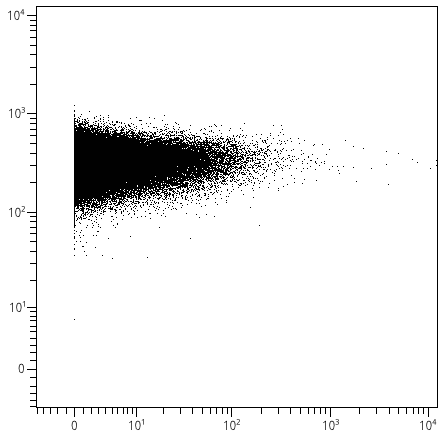

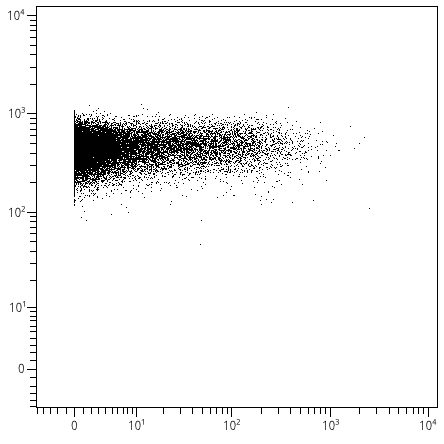

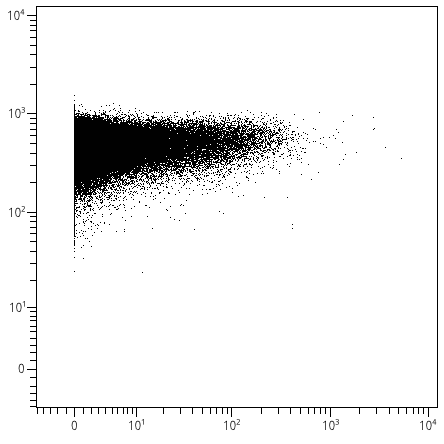

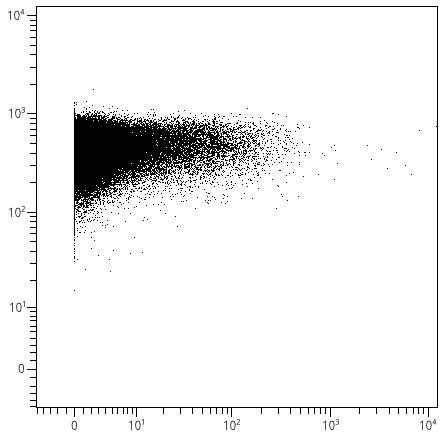


**regulatory T cells**


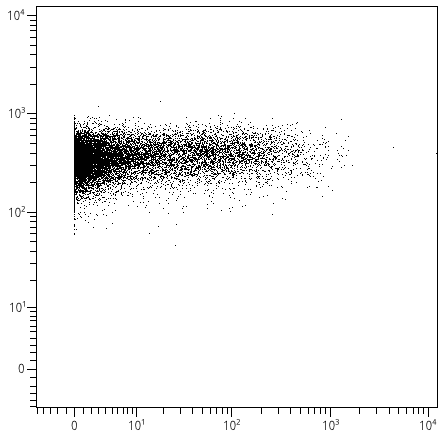


**CD4- CD8- T cells**


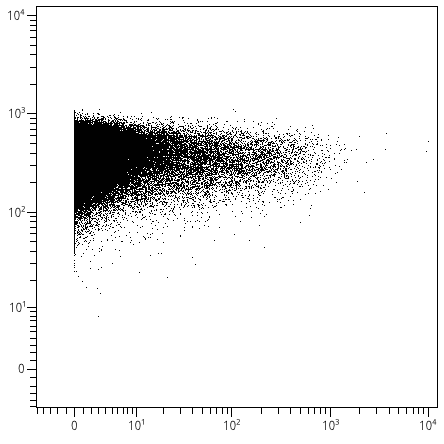


**CD4+**

**central memory**

**naive CD4^+^ T cells**

**CD4^+^**

**effector memory**

**CD8+**

**central memory**

**naive CD8^+^ T cells**

**CD8^+^**

**effector memory 1**

**CD8^+^**

**effector memory 2**

**regulatory T cells**

**CD4- CD8- T cells**


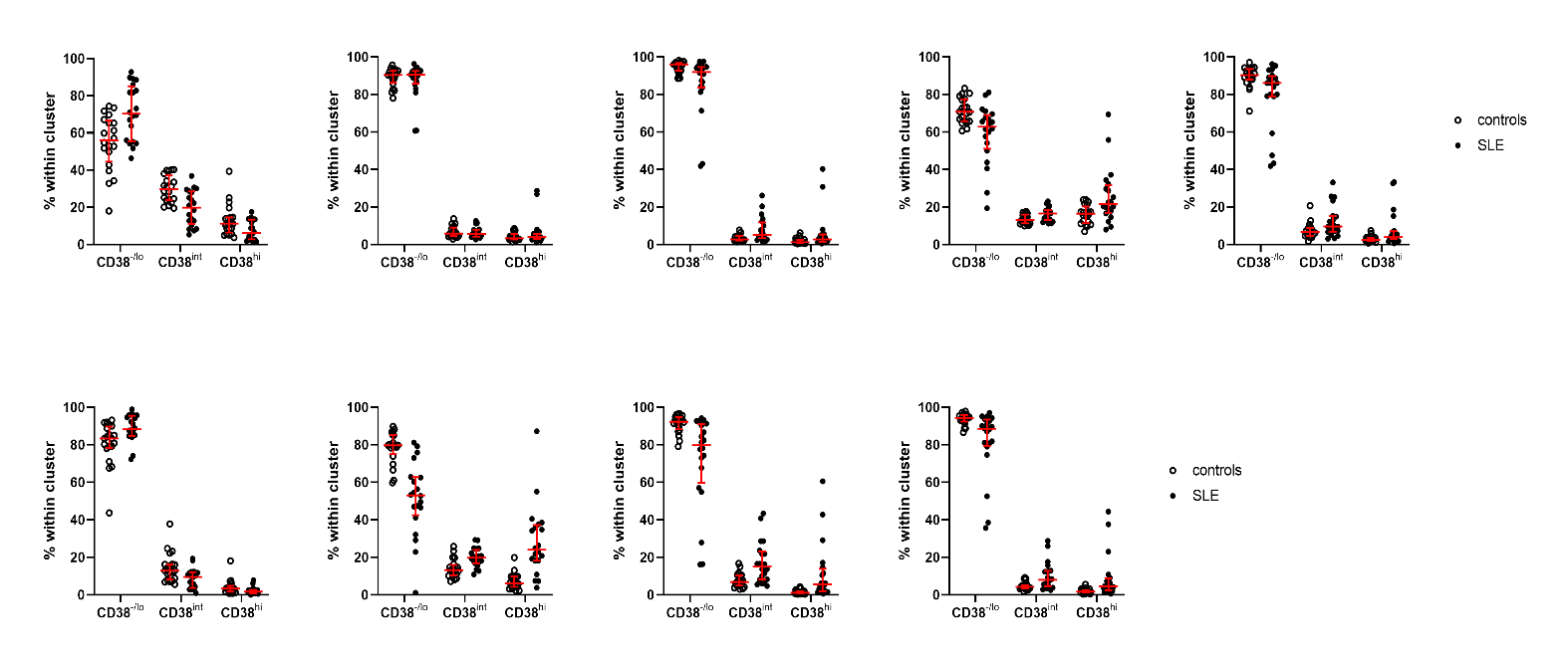


******

*****

******

*****

*****

*****

*****

*****

*****

******

*****

*****

*****

*****

*****

********

********

*******

*******

*******

*******

*******

*******

*******

**Figure S4.** Analysis of T cell subsets for their CD38 expression in patients with SLE and controls. This supplementary Figure extends Figure 3. (**A**) T -SNE map showing 15 T cell clusters generated by FlowSOM. CD4^-^ CD8^-^ T cell clusters (c6, c8, c9, c10, c11), CD4^+^ effector memory clusters (c4, c5) and CD8^+^ effector memory subsets (c7, c14) were merged for further analysis in Figure 3. Concatenated data of 20 healthy controls and 20 SLE patients are shown (**B**) T-SNE map colored by the expression of markers expressed by T cells . (**C**) T cell subsets were manually gated according to their CD38 expression, subsetting them into CD38^-/low^, CD38^int^ and CD38^hi^ expressing cells. Concatenated data of 20 healthy controls and 20 SLE patients are shown (**D**) The abundance of subsets gated in (**C**) was analyzed in healthy controls and SLE patients. Each dot represents the frequency of the indicated subset of one donor. Red lines indicate medians and interquartile ranges. Asterisks indicate significantly different frequencies in SLE patients vs controls revealed by Mann-Whitney testing (*, *p* < 0.05; **, *p* < 0.01; ***, *p* < 0.001; ****, *p* < 0.0001).

**B**

**A**


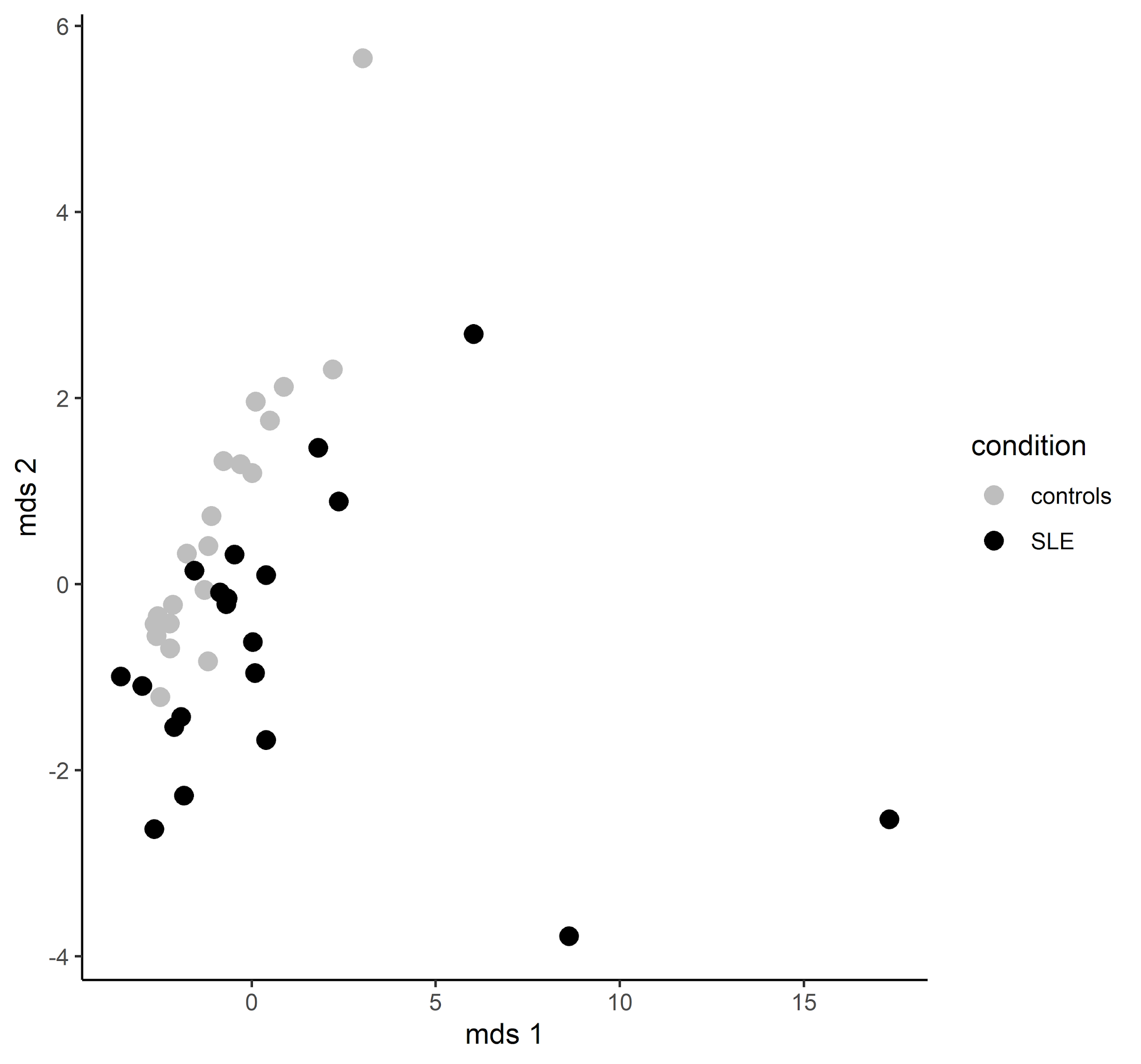


**controls**

**SLE**


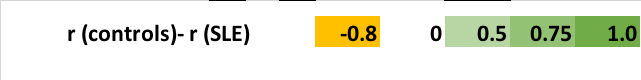

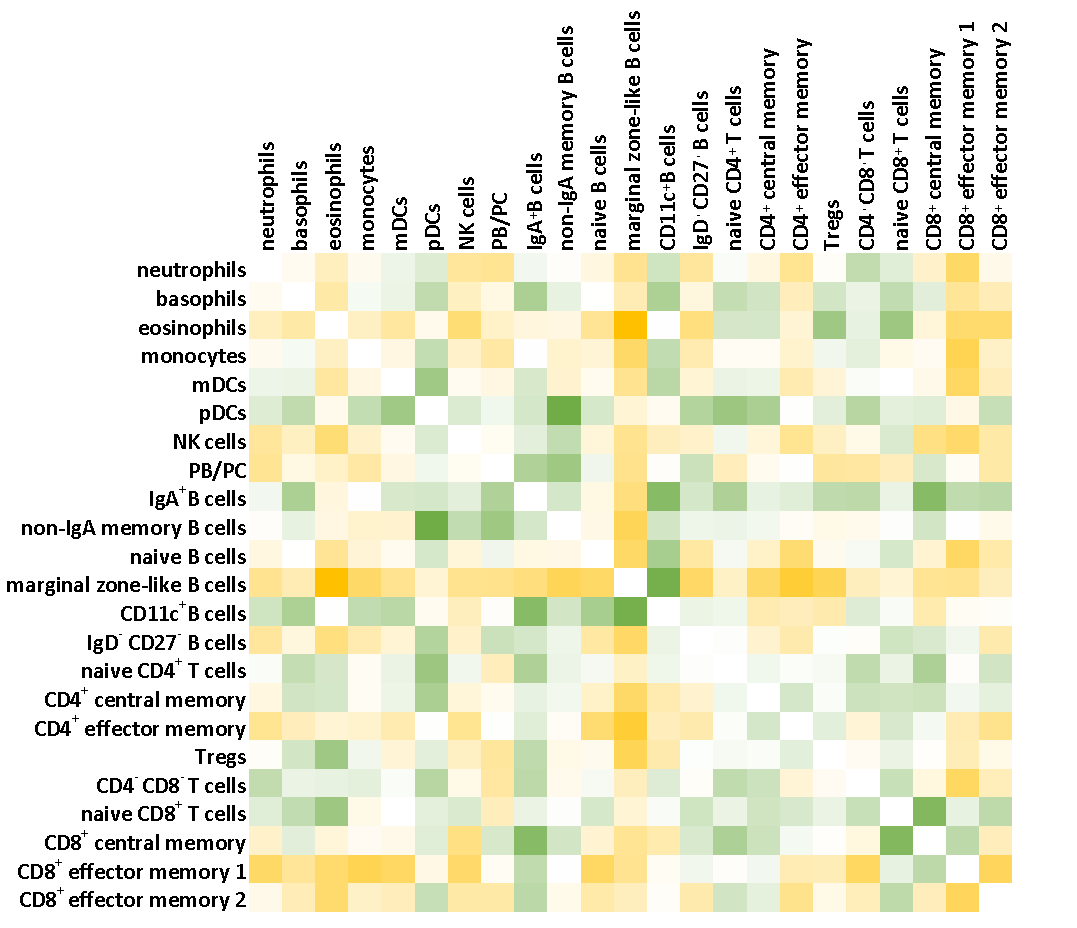


*

*

*

*

*

*

*

*

*

*

*

*

*

*

*

*

*

*

*

*

*

*

*

*

*

*

*

*

*

*

*

*

*

*

*

*

*

*

*

*

*

*

*

*

*

*

*

*

*

*

*

*

*

*

*

*

*

*

*

*

*

*

*

**Figure S5.** Differential correlation of CD38 expression between immune cell subsets in SLE and controls. This supplementary Figure extends Figure 5. (**A**) Spearman’s r values of SLE patients were subtracted from r values of controls (shown in Figure 5A,B) to highlight changes in correlation of CD38 expression levels between leukocyte subsets in the two groups. Asterisks indicate correlations that were considered significant in one group, but not found in the other, based on a *p* value of < 0.001. Yellow color indicates higher *r* values in SLE patients, green color indicates higher r values in controls. (**B**) Mean CD38 signal intensity values from all immune cell subsets analyzed in Figure 5 A,B were used to perform dimension reduction by multidimensional scaling.

**Table S1.** Mass Cytometry Antibodies and Details.

1. **Antibodies**

| isotope mass | metal | antibody target or reagent function | antibody clone | supplier | Cat no. | RRID | cell-surface (sf) /intracellular (ic) | used for global opt_SNE | used for B cell opt-SNE | used for T cell op t-SNE |
| --- | --- | --- | --- | --- | --- | --- | --- | --- | --- | --- |
| 89 | Y | CD15 | W6D3 | Biolegend | 323002 | AB_756008 | sf | x |  |  |
| 103 | Rh | mDOTA |  |  |  |  |  |  |  |  |
| 104 | Pd | barcoding |  |  |  |  |  |  |  |  |
| 105 | Pd | barcoding |  |  |  |  |  |  |  |  |
| 106 | Pd | barcoding |  |  |  |  |  |  |  |  |
| 108 | Pd | barcoding |  |  |  |  |  |  |  |  |
| 110 | Pd | barcoding |  |  |  |  |  |  |  |  |
| 113 | In | CD66b | REA306 | Miltenyi Biotec | 130-108-019 | AB_2658994 | sf | x |  |  |
| 115 | In | Siglec 8 | #837535 | R & D Systems | MAB7975 |  | sf | x |  |  |
| 140 | Ce | CD14 | RMO52 | Beckman Coulter | custom order |  | sf | x |  |  |
| 141 | Pr | CD127 | A019D5 | Biolegend | 351302 | AB_10718513 | sf | x |  | x |
| 143 | Nd | CD19 | BU12 | DRFZ | n.a. |  | sf | x | x |  |
| 145 | Nd | CD4 | RPAT4 | Biolegend | 300502 | AB_314070 | sf | x |  | x |
| 146 | Nd | CD45RO | UCHL1 | DRFZ | n.a. |  | sf | x | x | x |
| 147 | Sm | CD20 | Rituximab | Charité | n.a. |  | sf | x | x |  |
| 148 | Nd | IgA | IS11-8E10 | Miltenyi Biotec | 130-093-073 | AB_1036150 | sf | x | x |  |
| 149 | Sm | Syk | 4D10.2 | Fluidigm | 3149020B |  | ic | x | x |  |
| 151 | Eu | CD123 | 6H6 | Biolegend | 306002 | AB_314576 | sf | x |  |  |
| 152 | Sm | CD45RA | 4G11 | DRFZ | n.a. |  | sf | x | x | x |
| 154 | Sm | CD1c | AT5-8E7 | Miltenyi Biotec | 130-108-032 | AB_2661165 | sf | x | x | x |
| 155 | Gd | CD27 | 2E4 | Sanquin | M9185 |  | sf | x | x | x |
| 160 | Gd | CD11c | BU15 | Biolegend | 337202 | AB_1236381 | sf | x | x | x |
| 161 | Dy | CD7 | CD7-6B7 | Biolegend | 343102 | AB_1659214 | sf | x |  | x |
| 162 | Dy | IgM | MHM-88 | Biolegend | 314502 | AB_493003 | sf | x | x |  |
| 163 | Dy | CD197 | G043H7 | Biolegend | 353202 | AB_10945157 | sf | x | x | x |
| 167 | Er | CD38 | HIT2 | Biolegend | 303502 | AB_314354 | sf |  |  |  |
| 168 | Er | CD16 | 3G8 | Biolegend | 302002 | AB_314202 | sf | x | x | x |
| 169 | Tm | CD25 | MA-251 | Biolegend | 356102 | AB_2561752 | sf | x | x | x |
| 170 | Er | Siglec1 | 7-239 | Miltenyi Biotec | 130-108-017 | AB_2655556 | sf | x |  |  |
| 173 | Yb | IgD | IA6-2 | Biolegend | 348202 | AB_10550095 | sf | x | x |  |
| 174 | Yb | HLA-DR | L243 | DRFZ | n.a. |  | sf | x | x | x |
| 176 | Yb | CD56 | REA196 | Miltenyi | 130-108-016 | AB_2658728 | sf | x |  |  |
| 191 | Ir | DNA intercalator |  | Fluidigm | 201192A |  | ic |  |  |  |
| 193 | Ir | DNA intercalator |  | Fluidigm | 201192A |  | ic |  |  |  |
| 195 | Pt | CD3 | UCHT1 | DRFZ | n.a. |  | sf | x |  | x |
| 196 | Pt | CD8 | GN11 | DRFZ | n.a. |  | sf | x |  | x |
| 198 | Pt | CD45 | Hi30 | Biolegend | 304002 | AB_314390 | sf | x | x | x |
| 209 | Bi | CD11b | ICRF44 | Biolegend | 301302 | AB_314154 | sf | x |  | ? |
|  |  |  |  |  |  |  |  |  |  |  |
| n.a. - not applicable |  |  |  |  |  |  |  |  |  |  |

1. **Sample acquisition**

| Pool 1 | | | | | | | | |
| --- | --- | --- | --- | --- | --- | --- | --- | --- |
| patients | | | |  | **healthy controls** | | | |
| Code no | **gender** | **born** | **barcode** |  | **Code no** | **gender** | **born** | **barcode** |
| SLE_004_d0 | f | 1952 | **1** |  | ND036 | f | 1950 | **6** |
| SLE_020_d0 | f | 1960 | **2** |  | ND002 | f | 1960 | **7** |
| SLE_015 | f | 1972 | **3** |  | HC_006 | f | 1972 | **8** |
| SLE_012 | f | 1975 | **4** |  | HC_033 | f | 1976 | **9** |
| SLE_009 | m | 1983 | **5** |  | HC_012 | m | 1985 | **10** |
|  |  |  |  |  | *reference* | | | ***mDOTA*** |
|  |  |  |  |  |  |  |  |  |
| Pool 2 | | | | | | | | |
| patients | | | |  | **healthy controls** | | | |
| Code no | **gender** | **born** | **barcode** |  | **Code no** | **gender** | **born** | **barcode** |
| SLE_016 | f | 1965 | **1** |  | ND001 | f | 1966 | **6** |
| SLE_008 | f | 1979 | **2** |  | HC013 | f | 1978 | **7** |
| SLE_007 | f | 1983 | **3** |  | HC_028 | f | 1986 | **8** |
| SLE_017 | f | 1991 | **4** |  | HC_032 | f | 1993 | **9** |
| SLE_002 | m | 1993 | **5** |  | HC_017 | m | 1993 | **10** |
|  |  |  |  |  | *reference* | | | ***mDOTA*** |
|  |  |  |  |  |  |  |  |  |
| Pool 3 | | | | | | | | |
| patients | | | |  | **healthy controls** | | | |
| Code no | **gender** | **born** | **barcode** |  | **Code no** | **gender** | **born** | **barcode** |
| SLE_006 | f | 1983 | **1** |  | HC_030 | f | 1986 | **6** |
| SLE_010 | f | 1988 | **2** |  | HC_020 | f | 1986 | **7** |
| SLE_011 | f | 1985 | **3** |  | HC_029 | f | 1985 | **8** |
| SLE_018 | f | 1975 | **4** |  | HC009 | f | 1976 | **9** |
| SLE_021 | f | 1993 | **5** |  | HC008 | f | 1993 | **10** |
|  |  |  |  |  | *reference* | | | ***mDOTA*** |
|  |  |  |  |  |  |  |  |  |
| Pool 4 | | | | | | | | |
| patients | | | |  | **healthy controls** | | | |
| Code no | **gender** | **born** | **barcode** |  | **Code no** | **gender** | **born** | **barcode** |
| SLE_022 | f | 1993 | **1** |  | HC_023 | f | 1993 | **6** |
| SLE_019_d0 | f | 1975 | **2** |  | HC_019 | f | 1976 | **7** |
| SLE_003_d0 | f | 1967 | **3** |  | ND017 | f | 1967 | **8** |
| SLE_014_d0 | f | 1983 | **4** |  | ND025 | f | 1984 | **9** |
| SLE_001_d0 | f | 1976 | **5** |  | ND038 | f | 1971 | **10** |
|  |  |  |  |  | *reference* | | | ***mDOTA*** |

1. **Barcoding Scheme**

| **barcode no.** | **104Pd** | **105Pd** | **106Pd** | **108Pd** | **110Pd** | **103Rh** |
| --- | --- | --- | --- | --- | --- | --- |
| **1** | x | x |  |  |  |  |
| **2** | x |  | x |  |  |  |
| **3** | x |  |  | x |  |  |
| **4** | x |  |  |  | x |  |
| **5** |  | x | x |  |  |  |
| **6** |  | x |  | x |  |  |
| **7** |  | x |  |  | x |  |
| **8** |  |  | x | x |  |  |
| **9** |  |  | x |  | x |  |
| **10** |  |  |  | x | x |  |
| **reference** |  |  |  |  |  | x |

**Table S2.** Flow Cytometry Antibodies.

| **Target** | **Fluorochrome** | **Clone** | **Company** | **Catalogue Number** | **RRID** |
| --- | --- | --- | --- | --- | --- |
| IgD | FITC | IA6-2 | BD Biosciences | 555778 | AB_396113 |
| CD38 | APC | IB6 | Miltenyi Biotec | 130-092-261 | AB_871666 |
| CD27 | PE | O323 | Biolegend | 302808 | AB_314300 |
| CD24 | PerCP/Cy5.5 | ML5 | Biolegend | 311116 | AB_10960741 |
| HLA-DR | V500 | G46-6 | BD Biosciences | 561224 | AB_10563765 |
| CD3 | APC/Cy7 | SK7 | Biolegend | 344818 | AB_10645474 |
| CD14 | APC/Cy7 | 63D3 | Biolegend | 367108 | AB_2566710 |
| CD19 | APC/Cy7 | HIB19 | Biolegend | 302218 | AB_314248 |
| CD19 | V450 | HIB19 | Biolegend | 560353 | AB_1645564 |
| CD14 | FITC | 63D3 | Biolegend | 325604 | AB_830677 |
| CD16 | FITC | 3G8 | Biolegend | 302006 | AB_314206 |
| CD16 | Pacific Blue | 3G8 | Biolegend | 302032 | AB_2104003 |
| CD56 | PE/Cy7 | HCD56 | BD Biosciences | 318318 | AB_604107 |
| SIGLEC1 | PE | 7-239 | Biolegend | 346004 | AB_2189029 |
